# Distinct co-expression networks using multi-omic data reveal novel interventional targets in HPV-positive and negative head-and-neck squamous cell cancer

**DOI:** 10.1038/s41598-018-33498-5

**Published:** 2018-10-15

**Authors:** Raquel L. Costa, Mariana Boroni, Marcelo A. Soares

**Affiliations:** 1 Programa de Oncovirologia, Instituto Nacional de Câncer, Rio de Janeiro, Brazil; 2 Bioinformatics and Computational Biology Lab, Instituto Nacional de Câncer, Rio de Janeiro, Brazil; 3 Department of Genetics, Universidade Federal do Rio de Janeiro, Rio de Janeiro, Brazil

## Abstract

The human papillomavirus (HPV) is present in a significant fraction of head-and-neck squamous cell cancer (HNSCC). The main goal of this study was to identify distinct co-expression patterns between HPV+ and HPV− HNSCC and to provide insights into potential regulatory mechanisms/effects within the analyzed networks. We selected cases deposited in The Cancer Genome Atlas database comprising data of gene expression, methylation profiles and mutational patterns, in addition to clinical information. The intersection among differentially expressed and differentially methylated genes showed the negative correlations between the levels of methylation and expression, suggesting that these genes have their expression levels regulated by methylation alteration patterns in their promoter. Weighted correlation network analysis was used to identify co-expression modules and a systematic approach was applied to refine them and identify key regulatory elements integrating results from the other omics. Three distinct co-expression modules were associated with HPV status and molecular signatures. Validation using independent studies reporting biological experimental data converged for the most significant genes in all modules. This study provides insights into complex genetic and epigenetic particularities in the development and progression of HNSCC according to HPV status, and contribute to unveiling specific genes/pathways as novel therapeutic targets in HNSCC.

## Introduction

Head-and-neck squamous cell carcinoma (HNSCC) is a heterogeneous malignancy which accounts for approximately 300,000 deaths each year worldwide^1,2^. Smoking, alcohol, and infections by high-risk human papillomavirus (HPV) are among the main risk factors for the development of the disease. The incidence of HPV-associated HNSCC is around 25% of the reported cases worldwide, with an even higher proportion of oropharyngeal cancer, and a predominance of infection by HPV-16 among those cases^3–6^.

The development and progression of HNSCC occur by molecular deregulation events in many levels, including the accumulation of somatic mutations and changes in methylation profiles. Both those events result in differences in gene expression levels and downstream signaling pathways. In general, patients diagnosed with HPV+ HNSCC have a better prognosis (regardless of the treatment strategies) compared with the patients without HPV (HPV−) in the same anatomical site^7–9^. Although the molecular mechanisms involved in those difference are not fully understood, mutations in the *TP53* gene are massively more detected in HPV− compared to HPV+ tumors^10–12^.

With the advancement of high-throughput technologies, such as next-generation sequencing (NGS), efforts have been made to identify molecular characteristics that differentiate the profiles of HPV+ and HPV− HNSCC. Studies involving gene expression profiles have identified potential marker genes within each context. Masterson *et al*.^13^ identified markers of early-stage HPV+ oropharyngeal squamous cell carcinomas. Wood *et al*.^14^ identified distinct immune signatures in tumor-infiltrating lymphocytes (TILs), more specifically in B-cells, related to the adaptive immune response against HPV in those tumors. Gene expression involving microarray technology in HPV+ *versus* HPV− HNSCC has also been studied^15,16^. Other studies considered differences in methylation profiles. Esposti *et al*.^17^, for example, identified novel epigenetic signatures of HPV infection in HNSCC independent of the anatomical site. Studies involving more than one omic are increasing in the recent literature. Seiwert *et al*.^18^ used mutation and copy-number variation data to find unique mutations and aberrations in HPV+ HNSCC. Characterization of HNSCC subgroups using copy number alteration and transcriptome data were used in some studies^19,20^. The Cancer Genome Atlas (TCGA) consortium conducted a large study containing multi-platform and different types of tumors, including HNSCC. In 2015 the consortium carried out a comprehensive characterization of HNSCC samples including the identification of their HPV status^12^. In gene interaction networks, multi-layer integration is essential in the construction and functional understanding of the connections between genes at multiple levels^21^. With advances in research such as the TCGA mentioned above and other multi-omic repositories, it becomes possible to analyze a diversity of tumors through different platforms and technologies^22^.

In the present study, we have used HNSCC multi-omic data from the TCGA to explore the differences between gene co-expression networks of HPV+ and HPV− disease profiles. We first collected genes with significant differences in promoter methylation and gene expression profiles for each stage of the disease (Differentially Methylated Genes – DMG – and Differentially Expressed Genes – DEG –, respectively). The intersection among DMG and DEG showed the negative correlations between the levels of methylation and expression, suggesting that these genes have their expression levels regulated by methylation alteration patterns in their promoter. Based on global gene expression patterns, we applied Weighted Correlation Network Analysis (WGCNA) to identify gene modules associated with HPV status, followed by a computational strategy pipeline designed by us to refine the modules and build the networks for specific HPV profiles. In our results, the networks significantly associated with HPV statuses showed different connection patterns and brought new insights into mechanisms associated with HPV+ HNSCC. To our knowledge, this is the first study to conduct a gene network reconstruction via the integration of multi-omic sets for HPV+ and HPV− HNSCC.

## Results

### Gene expression profiles are influenced by methylation status in HPV+ and HPV− HNSCC

**Figure 1:**
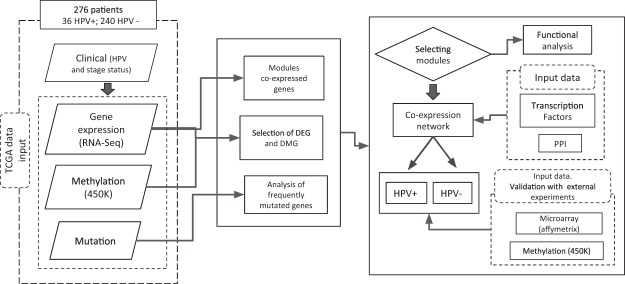
Flow diagram of the methodology applied to this study. The representation includes dataset preparation (dashed boxes), processes and analysis (middle and right panels, solid boxes).

The datasets studied were preprocessed and analyzed using the flowchart represented in Fig. 1. The preprocessing TCGA dataset for RNA-Seq level-3 resulted in 20,502 analyzed genes. For DNA methylation level-3, the dataset resulted in 14,861 analyzed genes. Two hundred and twenty-three DEG and 359 DMG were selected when comparing HPV+ and HPV− tumor samples (Supplementary Table 1). For methylation, only probes corresponding to the TSS200 annotation, following the strategy described in subsection *Omics datasets and preprocessing* were considered. Genes were selected using the limma package^23^ with restrictive parameters (FDR-adjusted p-value ≤ 0.01, absolute-logFC ≥ 4 and absolute-logFC ≥ 2 for expression and methylation levels, respectively) and evaluated for differences of HNSCC with HPV+ *versus* HPV− profiles within each disease stage (I–IV; Supplementary Fig. 1A) Among the studied genes, only a few remained differentially selected in most or all disease stages. Only six DEG were selected in all disease stages, while no DMG was common across disease stages.

**Figure 2:**
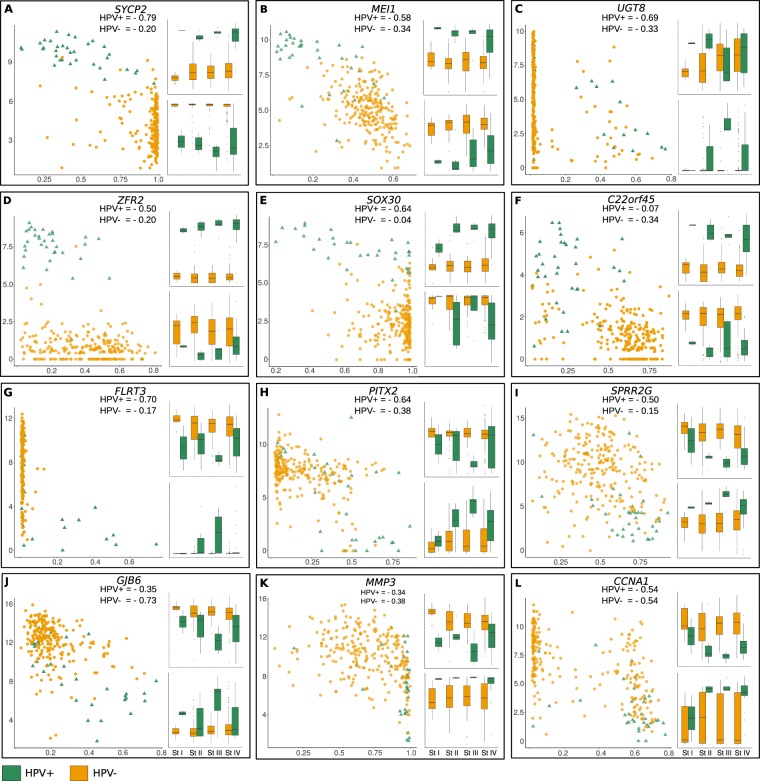
Negative correlation between gene expression and promoter methylation levels of genes doubly selected (**A**–**L**). For each gene, a scatter plot shows the correlation among methylation (*x*-axis) and expression levels (*y*-axis) for each profile (yellow circles for HPV− and green triangles for HPV+ samples). In each inset, the expression (upper panels) and the methylation levels (lower panels) are compared for each tumor stage (I to IV), using the same color codes for HPV+ and HPV− statuses.

The overlapping between DMG and DEG resulted in 14 genes which were doubly differential (Supplementary Fig. 1B). For this selection, a Pearson’s correlation (PC) was carried out between the expression and the methylation values (Fig. 2A–L). The *PNLDC1* and *CNTN1* genes were excluded from subsequent analysis due to their similar correlation profiles in both HPV+ and HPV− samples and both were differentially selected only in early stages, for which a limited number of samples was available for analysis. For all 12 genes evaluated, we found PC values < 0, showing a negative correlation between the two parameters. For seven genes (58.3%), the PC obtained for HPV+ HNSCC were higher than for HPV−. Our results are consistent with the knowledge of methylated promoter regions negatively regulating gene expression levels. In HPV+ cases, the *SYCP2*, *MEI1*, *UGT8*, *ZFR2* and *SOX30* genes were overexpressed when compared to the HPV− cases, an observation that was coupled with a decreased promoter methylation profile in the former (Fig. 2A–E). Conversely, the *FLRT3*, *PITX2* and *SPRR2G* genes were underexpressed in HPV+ cases compared to the HPV− cases (Fig. 2G–I). In those cases, a stronger negative correlation was seen in the HPV+ cases. On the other hand, the *GJB6* gene also exhibited underexpression in HPV+ cases, but a stronger negative correlation (rho = −0.73) in the HPV− cases. As expected, for those four latter genes, a consistent stronger promoter methylation was observed in the HPV+ cases (Fig. 2G–J).

### Gene modules were significantly associated with HPV status

In parellel to identifying DEG between HPV+ and HPV− HNSCC with high statistical confidence, we have also constructed gene co-expression networks using the WGCNA approach. This method calculates correlations among genes across samples and applies a power function to determine the connection strengths between genes resulting in a scale-free network^24,25^. Due to computational time, we used the 8,000 most variant genes regarding the median absolute deviation in expression profiles, which resulted in seven identified modules (Supplementary Fig. 2). The *minimum module* size was 20 and the *pickSoftThreshold* was 4 (Supplementary Fig. 3). The modules are referred to by their color labels in a hierarchical cluster dendrogram (Supplementary Fig. 2).

**Figure 3:**
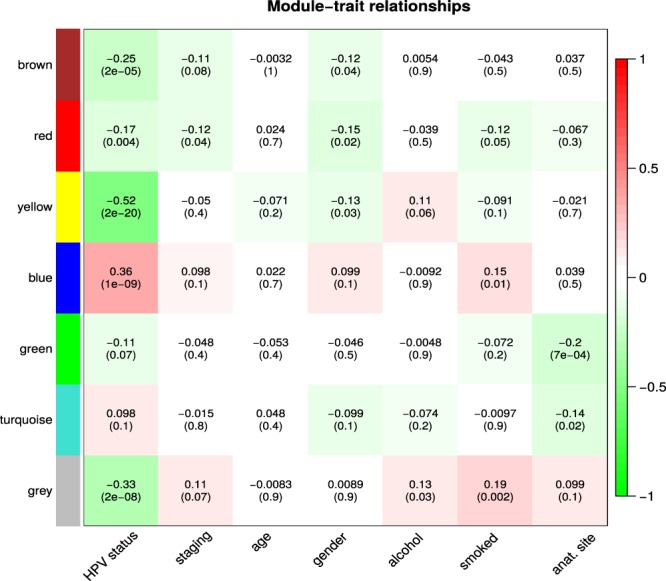
Co-expression genes modules and their relationship with studied traits. Matrix showing the correlation of the color-coded modules as generated with WGCNA (rows) with studied traits (columns). Cell contents display the correlation coefficients and p-values (in parentheses). Correlation coefficients were color-coded according to the heat index from red to green depicted at the vertical bar at the right to the graph.

Figure 3 depicts the correlations of the module eigengenes with the traits including ‘HPV status’, ‘staging’, ‘age’, ‘gender’, ‘alcohol’, ‘smoked’, and ‘anatomical site’. Three modules were found significantly associated with the HPV status (absolute correlation ≥0.25 and p-value ≤ 0.001), *Blue*, *Yellow* and *Grey*. The module membership (MM) *versus* gene significance (GS) plot for these modules and the borderline module *Brown* are shown in Supplementary Fig. 4. Despite candidate genes with no distinct module assignment were grouped in the *Grey* module, we have decided to include this module to subsequent analysis of the networks due to its significant association with HPV status. Therefore, the *Blue*, *Yellow* and *Grey* modules were further studied. We also computed the hierarchical clustering of the expression and methylation data of the samples concerning HPV status or disease staging using the ‘flashcluster’ function of WGCNA, but no clear clustering was observed (Supplementary Fig. 5).

### The *Blue*, *Yellow* and *Grey* gene modules result in distinct networks according to HPV status

**Figure 4:**
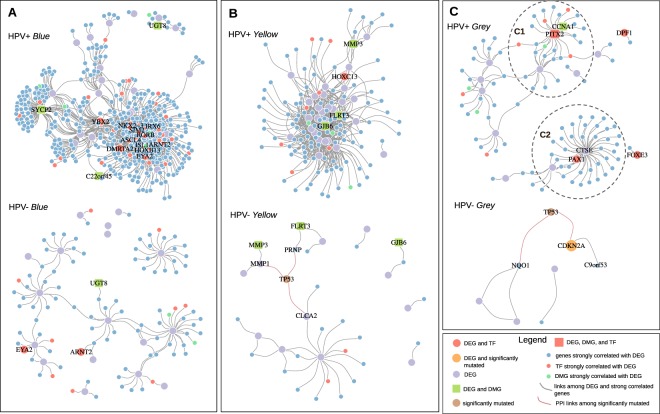
Co-expression networks among the modules with significantly different profiles between HPV+ and HPV− HNSCC cases. (**A**) *Blue* module. (**B**) *Yellow* module. (**C**) *Grey* module. In all modules, gene classifications are shape- and color-coded according to the legend at the lower right inset of the Figure. Links between DEG and strongly correlated genes and also those linking significantly mutated genes with genes through protein-protein interactions are also color-coded according to the legend of the Figure.

In general, the modules built by WGCNA contain a large number of genes when global expression data are used. As a consequence, some genes can be randomly associated with a specific phenotype. Thus, it is fundamental to identify relevant genes in the network, also known as hub genes, which are more likely to represent robust markers of specific phenotypes. In our approach, we used the previously selected DEG to guide the choice of hubs. For each selected module, we divided the samples by HPV status. We then computed the correlations in each status using all genes in each module (Spearman’s rank correlation coefficient). The genes selected in each module by HPV status were considered when these genes were DEG or when they were highly correlated with DEG. We applied a correlation threshold of ≥0.65 and applied a p-value threshold of ≤0.01 for both HPV− and HPV+ networks. In addition, we characterized the transcription factor genes (TF), doubly DEG/DMG, singly DMG and significantly mutated genes that engage in known protein-protein interactions (PPI) with present genes in each network (Fig. 4). Of the 12 double DEG/DMG genes considered for analysis (see above), eight appeared in one of the three modules kept for further analysis.

The networks were differentially connected according to HPV status (Table 1). All three *Blue*, *Yellow* and *Grey* modules had more densely connected networks in the HPV+ compared to HPV− cases, as measured both by the number of nodes and of edges (Table 1). In the HPV+ *Blue* network, the *SYCP2* (synaptonemal complex protein 2) is much more densely connected to other genes when compared to the HPV− network (Fig. 4A). The *C22orf45* gene, which showed few connections in the HPV+ *Blue* network, was not even evidenced in the HPV− counterpart, since no connections were established (Fig. 4A). Concerning TF genes, there are also stronger network connections, and a higher number of TF genes involved, when the HPV+ network is compared to the HPV− counterpart. Most TF genes in the HPV+ network are connected into a single high-density cluster, which is not seen in the HPV− network. Also of note, the *YBX2* is a TF that appears only in the HPV+ network, and connects the *SYCP2* hub to that high-density TF hub. It is worth mentioning that all genes visualized in this network (including the TFs) are overexpressed in HPV+ tumors.

**Table 1:** Connection metrics of co-expression networks of different modules in HPV+ and HPV− cases.

Module	HPV+	HPV−
*Blue*		
nodes	539	112
edges	2633	114
*Yellow*		
nodes	145	36
edges	640	34
*Grey*		
nodes	127	8
edges	111	7

The *Yellow* modules (Fig. 4B) depict genes that are generally overexpressed in HPV− compared to HPV+ tumors. In this set, *MMP3*, *FLRT3* and *GJB6* were doubly selected (in expression and methylation analyses) and more tightly connected in HPV+ tumors, denoting a concerted downregulated pathway. The *HOXC13* TF is also underexpressed in HPV+ tumors, and likely plays an important role in the connection of the pathways encompassing those genes.

The *Grey* modules (Fig. 4C) encompass genes that were not consistently clustered into any of the modules characterizing definite co-expression profiles. However, in the HPV+ network, specific co-expression gene sub-networks can be retrieved that show under or overexpression compared to HPV− tumors. Genes placed in central hubs of these two sub-networks can be visualized in the C1 and C2 inset circles of Fig. 4C, respectively. TFs which are DEG and/or DMG and involved in the control of these sub-networks include *PITX2* in C1 (underexpressed in HPV+ tumors) and *PAX1* in C2 (overexpressed in those tumors).

The original TCGA study on HNSCC has characterized that mutated genes were significantly more abundant in HPV− compared to HPV+ tumors^12^. Two of the top three significantly mutated genes (*TP53* and *CDKN2A*; Supplementary Fig. 6A,B) were integrated into the gene networks described above and had their locations and relationships visualized in the HPV− networks shown in Fig. 4B,C (bottom panels). *TP53* appears in two of the HPV− modules (*Yellow* and *Grey*), while *CDKN2A* appeared only in the *Grey* module, as it is also a DEG in that case. In the HPV− *Yellow* module, *TP53* appears connected with *MMP1*, *CLCA2* and *PRNP* (Fig. 4B, bottom panel). In the *Grey* module, *TP53* evidences a connection with *CDKN2A* and with *NKO1*, while *CDKN2A* itself is additionally associated with *C9orf53* (Fig. 4C, bottom panel).

### Enrichment functional analysis highlights specific HPV+ and HPV− biological pathways

To further explore the possible role of the gene modules and networks identified in our analyses of HNSCC with distinct HPV statuses, we performed enrichment analysis with Gene Ontology (GO) – Biological Process (BP), the Kyoto Encyclopedia of Genes and Genomes (KEGG) and the Molecular Signatures Database (MSigDB). The hypergeometric analysis was conducted with FDR-adjusted p-value ≤ 0.05. We captured the enriched functions of the identified modules with the R ‘clusterProfiler’ package. The main results are shown in Table 2. We identified pathways associated with cell fate specification and glycolysis/gluconeogenesis in the *Blue* HPV+ module. In contrast, genes of the HPV+ *Yellow* module were downregulated in the overrepresented biological processes of epidermis development, negative regulation of epithelial cell proliferation and keratinocyte differentiation. Finally, processes and pathways involving dendritic spine morphogenesis and lysosome degradation were overrepresented in HPV+ tumors, while cellular ketone metabolism and aging were underpinned in HPV− tumors (Table 2).

**Table 2:** Biological processes, pathways and molecular signatures significantly overrepresented in the *Blue*, *Yellow* and *Grey* modules according to HPV status (FDR-adjusted p-value ≤ 0.05).

Module	Status	Category	Id	Description	p-value^1^	Genes symbol
*Blue*	HPV+	GO (BP)	GO:0001708	cell fate specification	0.0285	*ISL1*, *EYA2*, *DMRTA2*, *FOXA1*, *LMO4*, *FZD7*, *POU5F1*, *FGF2*, *CDON*, *SOX2*
*Blue*	HPV+	KEGG	hsa00010	Glycolysis/Gluconeogenesis	0.026	*ALDH3A1*, *ADH6*, *ALDOB*, *ACSS1*, *ENO2*, *LDHC*, *ADH7*
*Blue*	HPV+	KEGG	hsa00564	Glycerophospholipid metabolism	0.026	*MBOAT1*, *PPAPDC1B*, *CHKA*, *CHPT1*, *DGKB*, *PLA2G6*, *ETNK2*, *DGKE*
*Blue*	HPV+	KEGG	GO:0001708	Glycine, serine and threonine metabolism	0.033	*CHDH*, *GNMT*, *PHGDH*, *AMT*, *CBS*
*Blue*	HPV+	KEGG	hsa04550	Signaling pathways regulating pluripotency of stem cells	0.033	*ISL1*, *FZD7*, *FZD8*, *LIFR*, *POU5F1*, *FGF2*, *FGFR2*, *PAX6*, *SOX2*
*Blue*	HPV+	MSigDB C6	—	RICKMAN HEAD AND NECK CANCER A	3.5e-09	*GABRP*, *MAP7D2*, *CLDN3*, *CLDN10*, *ARNT2*, *STAR*, *LIFR*, *C8orf4*, *C11orf93*, *FAM71E1*, *MYB*, *PARM1*, *STXBP6*, *CYP4X1*, *ZIC2*, *OLFM1*, *TMSB15A*, *NRCAM*, *TSPAN8*
*Blue*	HPV+	MSigDB C6	—	PYEON HPV POSITIVE TUMORS UP	9.7e-07	*MAP7D2*, *BTNL9*, *TCAM1P*, *SYCP2*, *ABCA17P*, *GLS2*, *IL17RB*, *ZSCAN16*, *CDK3*, *MYB*, *TM7SF3*, *CENPK*, *ANKRD36B*, *KIF15*, *ZNF238*, *SYNGR3*
*Yellow*	HPV+	GO (BP)	GO:0008544	epidermis development	9.2e-08	*CASP14*, *SPINK6*, *EREG*, *KLK5*, *KRT75*, *SLITRK6*, *FABP5*, *KRT14*, *PTHLH*, *HOXC13*, *CYP26B1*, *POU3F1*, *BNC1*, *IL20*, *APCDD1*, *LAMC2*, *MYO5A*, *CST6*, *INHBA*, *CTSV*, *CDH3*
*Yellow*	HPV+	GO (BP)	GO:0043588	skin development	1.18e-04	*CASP14*, *SPINK6*, *EREG*, *KLK5*, *KRT75*, *KRT14*, *HOXC13*, *CYP26B1*, *POU3F1*, *IL20*, *ITGA6*, *APCDD1*, *MYO5A*, *INHBA*, *CTSV*, *CDH3*
*Yellow*	HPV+	GO (BP)	GO:0050680	negative regulation of epithelial cell proliferation	1.5e-02	*EREG*, *GJA1*, *EFNB2*, *XDH*, *CAV1*, *CTSV*, *CDK6*
*Yellow*	HPV+	GO (BP)	GO:0030216	keratinocyte differentiation	1.6e-0	*CASP14*, *SPINK6*, *EREG*, *KLK5*, *KRT75*, *KRT14*, *CYP26B1*, *POU3F1*, *IL20*, *CDH3*
*Yellow*	HPV+	MSigDB H	—	HALLMARK EPITHELIAL MESENCHYMAL TRANSITION	0.005	*MMP3*, *MMP1*, *PTHLH*, *AREG*, *GJA1*, *TNC*, *LAMC2*, *INHBA*, *TNFRSF12A*, *NT5E*
*Grey* (*C1*)	HPV+	GO (BP)	GO:0060997	dendritic spine morphogenesis	0.042	KALRN, NGEF, EPHB3
*Grey* (*C2*)	HPV+	KEGG	hsa04142	Lysosome	0.041	*CTSE*, *PSAPL1*
*Grey*	HPV−	GO (BP)	GO:0042180	cellular ketone metabolic process	0.0011	*AKR1C2*, *AKR1C3*, *NQO1*
*Grey*	HPV−	GO (BP)	GO:0007568	aging	0.0028	*CDKN2A*, *NQO1*, *TP53*
*Grey*	HPV−	MSigDB H	—	HALLMARK XENOBIOTIC METABOLISM	0.007	*AKR1C2*, *AKR1C3*, *NQO1*

^1^FDR-adjusted.

### External biological datasets provide significant congruence with predicted networks

**Figure 5:**
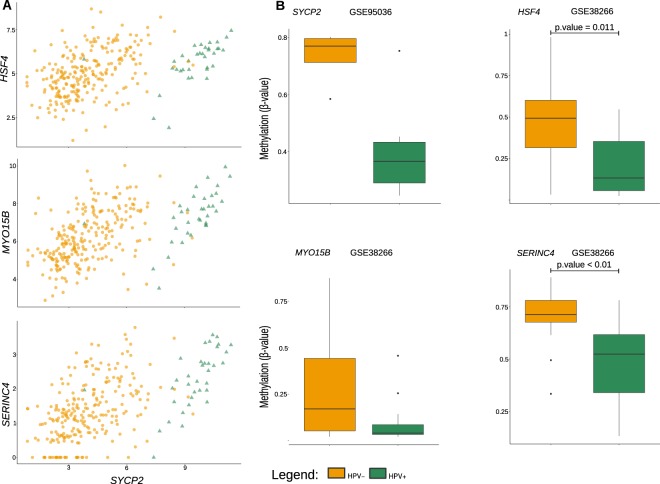
Comparison of gene expression and promoter methylation data between the TCGA data analyzed and those of independent biological experiments. (**A**) Scatter plots showing the correlation of expression levels of genes from TCGA data which were differentially methylated (*HSF4*, *MYO15B* and *SERINC4*; *y*-axes) with *SYCP2* (*x*-axes). The yellow circles represent HPV− samples, while the green triangles represent HPV+ counterparts. (**B**) Comparison of the methylation levels of the promoter region (TSS200) between HPV+ and HPV− samples in external high-throughput methylation experiments. The adjusted p-values that remained significant are shown.

We used three independent experiments using two different omics (gene expression and methylation), and distinct tisssue processing (fresh frozen *versus* FFPE) to check the concordance with our results. The genes selected from the microarray experiment by Pyeon *et al*.^26^ (GEO ID: GSE6791) were consistently similar to the modules and signals (up- or downregulated) of expression in our analyses obtained from the TCGA (Table 3). In the methylation datasets, we also found methylated genes in the promoter region (TSS200) similar to those of our analysis (Supplementary Table 2). The *SYCP2*, *PITX2* and *GJB6* genes, which were DEG and DMG in the TCGA analysis, were also DMG in the two independent datasets studied^17,27^. However, in the dataset from Esposti *et al*.^17^ (GEO ID: GSE95036) the significance of the test was lost when the p-values were adjusted (Supplementary Table 2). *SYCP2* and *PITX2* were also DEG in the Pyeon *et al*.^26^ dataset. The methylation levels of the *GJB6* and *PITX2* promoters in both independent methylation experiments are shown in Supplementary Fig. 7. When observing the connections in the *Blue* HPV+ network, the *HSF4*, *MYO15B* and *SERINC4* genes were strongly correlated with *SYCP2*. These genes were DMG in our analysis (Fig. 5A) and also found as DMG in Lechner *et al*.^27^ (GEO ID: GSE38226) (Fig. 5B).

**Table 3:** Differentially-expressed genes of the GSE6791^26^ external high-throughput experiment and congruence with our TCGA analysis.

Probe	Entrez Id	Symbol	FC^1^	P-value^2^	Signal	Module
1558856_at	63950	*DMRTA2*	1.91	0.006	$\uparrow$	*Blue*
204343_at	21	*ABCA3*	1.5	0.04	$\uparrow$	*Blue*
206546_at	10388	***SYCP2***	3.17	0.003	$\uparrow$	*Blue*
220378_at	6954	*TCP11*	1.48	0.004	$\uparrow$	*Blue*
228262_at	256714	*MAP7D2*	3.2	0.001	$\uparrow$	*Blue*
228434_at	153579	*BTNL9*	1.78	0.025	$\uparrow$	*Blue*
231164_at	650655	*ABCA17P*	2.89	<0.01	$\uparrow$	*Blue*
231517_at	440590	*ZYG11A*	1.47	0.036	$\uparrow$	*Blue*
233320_at	146771	*TCAM1P*	2.58	<0.01	$\uparrow$	*Blue*
237304_at	256126	*SYCE2*	1.65	0.013	$\uparrow$	*Blue*
244198_at	64901	*RANBP17*	1.39	0.032	$\uparrow$	*Blue*
205783_at	26085	*KLK13*	−1.55	0.032	$\downarrow$	*Brown*
206125_s_at	11202	*KLK8*	−1.72	0.007	$\downarrow$	*Brown*
206605_at	8909	*ENDOU*	−2.61	0.034	$\downarrow$	*Brown*
208539_x_at	6701	*SPRR2B*	−2.75	0.04	$\downarrow$	*Brown*
209792_s_at	5655	*KLK10*	−2.29	0.023	$\downarrow$	*Brown*
214549_x_at	6698	*SPRR1A*	−2.22	0.039	$\downarrow$	*Brown*
220620_at	54544	*CRCT1*	−2.59	0.047	$\downarrow$	*Brown*
220664_at	6702	*SPRR2C*	−2.86	0.048	$\downarrow$	*Brown*
233488_at	84659	*RNASE7*	−1.48	0.033	$\downarrow$	*Brown*
233586_s_at	43849	*KLK12*	−2.7	0.04	$\downarrow$	*Brown*
235272_at	374897	*SBSN*	−2.24	0.049	$\downarrow$	*Brown*
206561_s_at	57016	*AKR1B10*	−2.23	0.026	$\downarrow$	*Grey*
207039_at	1029	*CDKN2A*	3.46	<0.01	$\uparrow$	*Grey*
207366_at	3787	*KCNS1*	1.32	0.016	$\uparrow$	*Grey*
207558_s_at	5308	***PITX2***	−1.94	0.041	$\downarrow$	*Grey*
219263_at	79589	*RNF128*	−2.05	0.019	$\downarrow$	*Grey*
220325_at	54457	*TAF7L*	1.78	0.007	$\uparrow$	*Grey*
232604_at	84215	*ZNF541*	1.35	0.001	$\uparrow$	*Grey*
1556300_s_at	6492	*SIM1*	2.65	0.026	$\uparrow$	*Turquoise*
205551_at	9899	*SV2B*	1.96	0.029	$\uparrow$	*Turquoise*
207678_s_at	11063	***SOX30***	2.03	0.01	$\uparrow$	*Turquoise*
219753_at	10734	*STAG3*	2.26	<0.01	$\uparrow$	*Turquoise*
220507_s_at	51733	*UPB1*	2.09	0.008	$\uparrow$	*Turquoise*
229024_at	57484	*RNF150*	1.09	0.036	$\uparrow$	*Turquoise*
230011_at	150365	***MEI1***	1.46	0.033	$\uparrow$	*Turquoise*
233064_at	23217	***ZFR2***	4.23	<0.01	$\uparrow$	*Turquoise*
202345_s_at	2171	*FABP5*	−1.87	0.017	$\downarrow$	*Yellow*
205627_at	978	*CDA*	−2.23	0.03	$\downarrow$	*Yellow*
205767_at	2069	*EREG*	−2.37	0.028	$\downarrow$	*Yellow*
206165_s_at	9635	*CLCA2*	−1.84	0.044	$\downarrow$	*Yellow*

Genes were selected by moderated t-tests comparing HPV+ and HPV− samples. Modules are as indicated in Fig. 3.

^1^absolute-logFC ≥ 1; ^2^FDR-adjusted ≤ 0.05.

Genes in boldface are those also found as differentially-methylated in our analysis.

## Discussion

The heterogeneity of HNSCC with respect to the variety of anatomical sites and driving behavioral (alcohol, tobacco, hot beverages) and infectious (HPV) factors makes the identification of relevant therapeutic targets a challenging task^11,28,29^. In addition, the analysis of mono-omic data, i.e. from a single layer, provides only one dimension of a multifaceted scenario, and limited information about the possible molecular mechanisms involved in the disease. Genetic and epigenetic changes such as mutations and methylation patterns modulate gene expression levels of several genes. Although both result in the same phenotypic alteration (changes in expression levels), the genetic mechanisms involved and the adjacent gene interactions (gene networks) are different, an observation that can only be done with the analysis of multi-omic data^30–32^. In this sense, the analysis of data such as those available through the TCGA Consortium provides a unique opportunity to assess multi-layer molecular interactions in a feasible manner^22,33^. In the current study, we utilized HNSCC multi-omic data from TCGA in an attempt to more comprehensively understand gene co-expression networks and the putative roles of gene promoter methylation patterns and gene mutations associated with HPV+ and HPV− profiles through disease progression.

Our approach started with the identification of DEG between HPV+ and HPV− tumors. The genes involved in these networks appeared to vary significantly when we use data from different disease stages (I through IV) of both HPV statuses (Supplementary Fig. 1, Supplementary Table 1 and Fig. 2), indicating that their expression is modulated to different levels during the carcinogenic process. Despite the fact that most genes lost statistical significance in differential expression between HPV+ and HPV− tumors at one or more disease stages, a general trend could be observed that DEG maintained their patterns throughout the stages (i.e., being over or underexpressed in HPV+ compared to HPV− cases; Supplementary Table 1 and insets of Fig. 2). It is worth mentioning that data from a small number of cases were available for initial tumor stages, particularly from HPV+ cases, and we cannot exclude the possibility that such heterogeneity in the number of samples compromised the robustness of the differences observed herein. Analyses with larger numbers of cases are warranted in further studies to more precisely identify DEG throughout HNSCC stages. On the other hand, most of the converging expression results (i.e., lack of significant differential expression between HPV+ and HPV− cases) occurred in stage IV (data not shown). It is tempting to speculate that, at an advanced disease stage, the molecular processes converge between HPV+ and HPV− cases, being the virus a mere initiator of the carcinogenesis via distinct pathways. A similar scenario was observed when DMG were derived from the same data (Supplementary Table 1). Again, promoter gene methylation patterns differed between HNSCC stages comparing HPV+ and HPV− cases and no single gene differed significantly across all four stages between the two HPV statuses. These results indicate that the association between methylation and gene expression is stronger in HPV-infected HNSCC, and thus that epigenetic regulation appears to be pivotal during HPV infection of head-and-neck anatomical sites.

Gene co-expression modules and networks were constructed using global expression data (Fig. 3), and DEG were used as filters for refining those networks as described in Methods. In this sense, only genes that were DEG or directly interact with DEG in a linear positive fashion were plotted in module networks (Fig. 4). Three significantly supported modules (*Blue*, *Yellow* and *Grey*) were further investigated. The obtained gene networks differed between HPV+ and HPV− tumors within each module (Fig. 4), suggesting that HPV infection plays a unique role in HNSCC carcinogenesis, which involves a series of distinct molecular processes from the HPV− counterparts. To the best of our knowledge, very few studies (if any) tried to assess the composition of gene networks through disease progression and also how HPV influences that development. In all three modules studied, the HPV+ networks were much more densely connected and encompassed a larger number of significant nodes and edges compared to the HPV− counterparts (Fig. 4). Irrespective of the modulation provided by the presence of the virus (either gene overexpression or underexpression compared to the HPV− networks in the *Blue* or *Yellow* module, respectively), the networks suggest a fundamental role of HPV in hijacking and modulating specific biological processes within tumor cells.

We further integrated DMG and significantly mutated genes in the HPV+ and HPV− module networks by identifying those genes within the networks. Since the networks are composed of DEG, the DMG identified are necessarily doubly DEG and DMG. Despite these genes were essentially the same when comparing the HPV+ and HPV− networks within each module (with one or two exceptions in each module), their engagement in different interactions were noteworthy (compare HPV+ and HPV− modules in Fig. 4A–C). In the HPV+ networks, particularly in the *Yellow* module, DMG were more central in the networks and engaged in higher numbers of connections (compare HPV+ and HPV− in Fig. 4B). This is consistent with the observation that DEG in the *Yellow* module are repressed (underexpressed) in the HPV+ cases compared to HPV− counterparts. With respect to the mutated genes, three appeared significantly mutated in HPV− compared to HPV+ cases, *TP53*, *CDKN2A* and *FAT1* (Supplementary Fig. 6B). Of those three, only *CDKN2A* appeared in one of the modules (*Grey*), because it is also a DEG (Fig. 4C). On the other hand, *TP53* also showed relevant PPI with genes in the *Yellow* and *Grey* modules as evidenced through searches within StringDB, and was arbitrarily added to those two modules (Fig. 4B,C). As expected, all those occurrences took place in the HPV− networks, consistent with the fact that mutations in those genes were reported almost exclusively in subjects with HPV− status (see Supplementary Fig. 6A). Our results point to a fundamental, yet expected role of host gene mutations as primary drivers of carcinogenesis in HPV− samples, as opposed to an infectious agent driver in the case of HPV+ samples. Of note, mutations in the *PIK3CA* (phosphatidylinositol-4,5-bisphosphate 3-kinase catalytic subunit alpha) gene have been recently associated with HPV+ HNSCC^12,34^. We did not find such association, since those mutations were also present in HPV− tumors, and the difference between the two HPV statuses was not significant (19% of HPV− versus 36% of HPV+ cases), a comparison likely not conducted by TCGA in their report^12^.

Several genes could be retrieved from the gene co-expression networks obtained from the three modules which appear to have distinguishable importance in HPV+ and HPV− HNSCC. The *SYCP2* gene encodes the synaptonemal complex protein 2, a protein that is localized in chromosomal centromeres and responsible for the association of chromosomes with the synaptonemal complex, driving the prophase of meiosis^35^. *SYCP2* has been found overexpressed in HPV+ oropharyngeal cancers^13^, and similar results were found herein for HNSCC in general (Fig. 2A). It was also associated with cervical squamous cell carcinomas^36^. Of note, Peyon *et al*.^26^ have proposed that HPV+ cancers from distinct anatomical sites, specifically cervical cancers and HNSCC, share many upregulated genes and pathways, including the overexpression of testis-specific genes involved in meiosis such as *SYCP2*^26^. Aberrant expression of this gene in HPV+ cancers likely contribute to genomic instability and further oncogenic alterations, yet a specific interaction of viral products with *SYCP2* is yet to be elucidated.

Transcription factors were also overexpressed in the HPV+ *Blue* network, such as *YBX2*, *DMRTA2* and *EYA2* (Fig. 4A). Most of these TF have been described as overexpressed in different types of cancers, including ovarian, testis and breast cancer in the case of *YBX2*^37,38^, and breast cancer, lung cancer and acute myeloid leukemia in the case of *EYA2*^39,40^. *DMRTA2*, on the other hand, is also expressed in the spermatogenesis of the testis, and regulates the cyclin-dependent kinase CDKN2c^41^, in addition to maintaining neuroprogenitor cells in the cell cycle^42^. Although the specific role of HPV in upregulating these TF is unknown, gene silencing of *EYA2* significantly reduced viability, migratory capacity, and anchorage-independent growth of HPV16-transformed keratinocytes^43^. Moreover, our results point to a fundamental interaction of HPV with a defined network of genes that regulate gametogenesis in the testis and ovaries, a pathway that warrants further study for interventional approaches. Additional genes that are co-expressed with the abovementioned ones in a highly significant fashion, such as *MYO3A* (myosin IIIA), *IL17RB* (interleukin 17 receptor B) and *UBXN11* (UBX domain protein 11) (please see the complete network visualization described in the “Data Availability” section at the end of this report), and for which scarce information as related to carcinogenesis or HPV infection is available, are also attractive for further studies and as targets for intervention. According to the GO biological processes associated with the reconstructed HPV+ *Blue* network, cell fate differentiation and glucose metabolism appear to be major components (Table 2), consistent with gene upregulation that occurs during tumor development.

In the HPV+ *Yellow* network, two central genes were shown to be significantly underexpressed and more methylated compared to HPV− HNSCC, *GJB6* (gap junction protein beta 6, also known as connexin 30) and *FLRT3* (fibronectin leucine rich transmembrane protein 3) (Figs 2 and 4B). Furthermore, the *HOXC13* (homeobox C13) TF, a regulator of several genes during epithelial differentiation, and of which mutations were associated with pure hair and nail ectodermal dysplasia^44^, is also underexpressed in this HPV status. Conversely, *HOXC13* and *FLRT3*, among other genes seen in our *Yellow* networks, were found upregulated in HPV− OSCC^45^, in agreement with our results. Not surprisingly, all these genes have been associated with the expression and metabolism of gap junction proteins and keratins, as well as keratinocyte differentiation in epithelial cells, and appeared to be downregulated in HPV+ tumors. Other genes significantly associated with those are keratins 14 and 19 (*KRT14*, *KRT19*), *COL4A6* (collagen type IV alpha 6 chain) and *CLCA2* (chloride channel accessory 2), which are also involved in keratinocyte biology. These results are highlighted in the GO analysis for this network, which showed an enrichment in negative regulation of epithelial cell proliferation, keratinocyte differentiation, and skin and epidermis development (Table 2). The *MMP3* gene encodes the matrix metallopeptidase 3 and is generally associated with multiple steps of cancer development, invasion and metastasis^46^. Interestingly, this gene was also underexpressed in our HPV+ compared to the HPV− *Yellow* network (Fig. 4B). It is tempting to speculate that, in a scenario where most adhesion and gap junction molecules are already downregulated, upregulation of *MMP3* is not a *sine qua non* step towards tumor cell invasion and metastasis.

In the HPV+ *Grey* network, two genes were found underexpressed and hypermethylated compared to HPV− tumors, *PITX2* (paired like homeodomain 2) and *CCNA1* (cyclin A1) (Figs 2 and 4C). The first one is additionally a TF which has been implicated in muscle development. *PITX2* hypermethylation has been interestingly associated with better prognosis in HNSCC^47^ but with worse prognosis in breast cancer^48^. *PITX2* has also been shown to control the expression of *CCNA1* in a positive fashion^49^, which fits the relationships found in our network. Moreover, HPV-16 E7 has also been implicated in the mediation of *CCNA1* promoter methylation^50^. Conversely, *PAX1* (paired box 1) and correlated genes (Fig. 4C, inset C2) are overexpressed in HPV+ compared to HPV− tumors. One of these genes, the DEG *CTSE* (cathepsin E), is involved in the lysosome degradation pathway (KEGG, hsa04142). *CTSE* has been additionally recognized as a biomarker for the detection of pancreatic ductal adenocarcinoma^51^ and for gastric cancer^52^.

In the HPV− *Grey* module, no clear networks were formed, but underexpression of *CDKN2A* and its association with *TP53* were evident (Fig. 4C). Moreover, *TP53* and *CDKN2A* were significantly mutated in this network (Supplementary Fig. 6B). *CDKN2A* is a kinase implicated in the production of p16(INK4a) and p14(ARF), well-established tumor suppressors. Therefore, decreased expression of *TP53* and *CDKN2A* by inactivating mutations as seen in our data fits the scenario of HPV− induced carcinogenesis, where cellular genes are the major drivers of the process.

Our novel findings with the multi-omic integration of the HNSCC TCGA dataset were further confronted with three independent studies reporting experimental data acquisition from biological samples. One study derived gene expression data using microarray analysis^26^, a different method from the TCGA dataset, while the other two analyzed methylation profiles using either fresh frozen^27^ or FFPE^17^ samples. Independent of omics, methods or sample preparation protocols used, the data from those three studies converged siginificantly with our findings using TCGA. A comprehensive list of genes differentially expressed between HPV+ and HPV− tumors was found concordant between our study and that by Pyeon *et al*.^26^ (GEO: GSE6791) (Table 3). Of note, among the 12 simultanously DEG and DMG pointed out in our analysis (Fig. 2), five were represented in that list (boldfaced in Table 3). With respect to methylation, we found that six out of the 12 genes mentioned above were also DMG in the study by Lechner *et al*.^27^ (GEO: GSE38266). Conversely, *SYCP2*, a gene featured as DMG in the HPV+ *Blue* network in our analysis (Fig. 4A), was also evidenced when analyzing the data by Esposti *et al*.^17^ (GEO: GSE95036) (Table 3). Of note, three other genes intimately correlated with *SYCP2* for their DMG patterns in our analysis (*HSF4*, *MYO15B* and *SERINC4*) (Fig. 5), have also appeared in the data by Lechner *et al*.^27^, unveiling a central regulatory pattern in gene expression/methylation in HPV+ tumors. Two of the 12 DMG/DEG genes found in our HPV+ networks, *GJB6* and *PITX2*, emerged as DMG in both experimental methylation datasets^17,27^ (Supplementary Fig. 7), highlighting them as pivotal to the carcinogenesis of HPV+ tumors.

Overall, the results presented herein emphasize the importance of integrating different genomic data (as mRNA expression, DNA methylation and mutation patterns) to get a better understanding of the molecular mechanisms involved in the carcinogenesis and progression of HNSCC, an approach that can be applied to other tumor types. Even though the individual analysis of one biological level (mRNA) gives information associated with the disease, the integration with other biological levels is required to have a more comprehensive view from a functional perspective, allowing the identification of novel molecular targets unseen by mono-omic approaches.

## Methods

### Omics datasets and preprocessing

The multi-omic data of HNSCC were retrieved from The Cancer Genome Atlas (TCGA) database^22^ by selecting the datasets published in 2015^12^ which identified HPV-positive (HPV+) and HPV-negative (HPV−) cases, totalling a set of 279 patients with data of primary solid tumors (HPV+: (stage I) n = 2; (stage II) n = 6; (stage III) n = 5; (stage IV) n = 22. HPV−: (I) n = 12; (II) n = 44; (III) n = 40; (IV) n = 144). Using clinical data information, we grouped the samples by HNSCC staging, which excluded three patients for whom this information was absent. The resulting dataset for further analysis consisted of 240 HPV− and 36 HPV+ cases.

The gene expression dataset was composed of data generated in an Illumina HiSeq. 2000 RNA-Seq platform (level 3) using the preprocessed RNAseqV2 normalized count expression values based on RNA-Seq by Expectation-Maximization (RSEM). We performed a log-transformation log(1 + p) on the count expression values. Genes with a zero standard deviation were removed from the dataset.

The methylation dataset was determined using Infinium HumanMethylation450 BeadChip (450 K). In the methylation level 3 data, each probe (CpG site) is measured as the ratio (*β* value) of the signal of methylated probes with respect to the sum of methylated and unmethylated probes, which varied continuously from 0 to 1, values that indicate *unmethylated* and *fully methylated*, respectively. We removed cross-reactive, non-specific, single nucleotide polymorphisms (SNPs), chromosomes *X* and *Y* and probes with genomic coordinates set to zero. We also removed probes with more than 5% missing values across samples. In the remaining data, absent data were estimated using the weighted k-nearest neighbor (kNN) algorithm, with k = 10, as proposed by Troyanskaya *et al*.^53^ and implemented in the R ‘impute’ package. The raw data (*M* values) normalization was performed with the BMIQ method proposed by Teschendorf *et al*.^54^ and implemented in the Chip Analysis Methylation Pipeline (ChAMP)^55^. The analysis of DMG was performed with the defined promoter region, following the methodology used by Jiao *et al*.^56^. Briefly, the average value of the probes mapping within 200 bp of the transcription start site (TSS) was assigned to the gene. If no probes mapped within 200 bp of the TSS, we used the average value of probes mapping to the 1st exon of the gene. If such probes were also not available, we used the average value of probes mapping within 1500 bp of the TSS.

The somatic mutation data were obtained from the Mutation Annotation Format (MAF) files. MAF files provide baseline data for many downstream analyses identifying somatic mutations in cancers through various pipelines and sequencing platforms. MAF files provide baseline data for many downstream analyses identifying somatic mutations in cancers through various pipelines and sequencing platforms.

### Genes selected by differences among stages in expression and methylation data

We selected significant genes (False Discovery Rate, FDR-adjusted p-value ≤ 0.01) comparing each profile (HPV+ versus HPV−) for each HNSCC stage. For instance, HPV+ (stage I) vs HPV− (stage I), …, HPV+ (stage IV) vs HPV− (stage IV). Genes that were selected in at least one comparison were included in posterior analyses. We used this approach for the RNA-Seq dataset including absolute log Fold-Change (absolute-logFC) ≥ 4, resulting in Differentially Expressed Genes (DEG). For the methylation dataset, we used the same method but considering the absolute-logFC ≥ 2 for selecting the DMG. These analyses were achieved based on normalized datasets by the fitting of the linear model (for each probe or gene) followed by moderated t-tests implemented in the ‘limma’ package^23^. We overlapped the DEG and DMG to determine genes that were doubly selected. Next, we calculated the Pearson’s correlation (PC) between the methylation and expression values to those doubly selected genes, and considered those with rho values ≤ 0.5 as significant.

### Somatic mutation analysis

Somatic mutations from Whole Exome Sequencing (WXS) in HNSCC were downloaded in a MAF file. We performed Fisher’s exact test to detect the differentially mutated genes on all HPV+ versus HPV− profiles with the ‘maftools’ package^57^. Adjusted p-values ≤ 0.05, provided by the FDR analysis^58^, were considered significant.

### Co-expression modules via WGCNA

The analysis of the co-expression network modules was performed using the package Weighted Correlation Network Analysis (WGCNA)^59^, applying the *minimumModuleSize* = 20 and *mergingCutHeight* = 0.45. The similarity matrix was converted to a weighted adjacency matrix by raising it to the power of *β* to amplify the strong connections and penalize the weaker connections. Gene expression was summarized into the module eigengene (ME) as the first principal component (PC) of the entire module gene expression. ME values were then correlated with the various studied traits. The trait-associated mRNAs were then subjected to WGCNA^60^ for the identification of high co-expression modules, denoted as *M*. The clinical data used in the analysis was related with ‘HPV status’, ‘staging’, ‘age’, ‘gender’, ‘alcohol’, ‘smoked’, and anatomical site (‘anat. site’). A subset *M*′ of *M* is given by modules significantly associated with HPV status selected for posterior analysis (absolute correlation >0.25 and p-value ≤ 0.001).

### Refining modules and interactions networks

Due to the number of genes in high-throughput data, the resulting modules contain a large number of genes, with interconnections that might result from spurious correlations. In order to obtain a selective and restrictive set of genes involved in each profile, we filtered the nodes in HNSCC for HPV+ and HPV− phenotypes. For this, assuming we have *n* selected modules, each selected module ${M}_{i}^{^{\prime} }$ of $M^{\prime} \subseteq M$ is represented by

$${M}_{i}^{^{\prime} }=\langle {G}_{i},S\rangle ,{G}_{i}=\{{g}_{i1},\ldots ,{g}_{i|{G}_{i}|}\},S=\{{s}_{1},\ldots ,{s}_{|S|}\};\,1\le i\le n,$$

where *G*_*i*_ is a set of genes and *S* is the set of samples. We separated the modules in,

$${M}_{i}^{+}=\langle {G}_{i},{S}^{+}\rangle ,{S}^{+}=\{s\in S|statusHPV(s)=+\,\},$$

for HPV+ and

$${M}_{i}^{-}=\langle {G}_{i},{S}^{-}\rangle ,{S}^{-}=\{s\in S|statusHPV(s)=-\,\},$$

for HPV−. For each ${M}_{i}^{v}$, $0\le i\le n$ where $v\in \{\,+,-\,\}$,

$${C}_{i}^{v}=\{({g}_{iy},{g}_{iz})|1\le y,z\le |{G}_{i}|,\,\{{g}_{iy},{g}_{iz}\}\subset {G}_{i}\},\,{\rm{where}}\,{g}_{iy}\,{\rm{is}}\,{\rm{DEG}}\}.$$

We select the genes ${g}_{iy}$ which $cor{r}_{{S}^{v}}({g}_{iy},{g}_{iz})\ge 0.65$, $y > z$, $({g}_{iy},{g}_{iz})\in {C}_{i}^{v}$ and p-value ≤ 0.01.

The resulting networks were visualized with the ‘igraph’ package available in R CRAN^61^. DEG, DMG, doubly selected (DEG and DMG), transcription factors (TF) and significantly mutated genes were identified in the network. The TF data were obtained from the *TFcheckpoint* database^62^. To link the significantly mutated genes in the network, we used the protein-protein Interaction (PPI) associations from the STRING database^63^, with high confidence (score ≥ 0.7) selected.

### Gene Ontology and pathway-enrichment of the selected genes within modules

To identify the significant enrichment pathways, Gene Ontology (GO) terms^64,65^, KEGG^66^ and the Molecular Signature Database (MSigDb v.6.0)^67^ were used. The hypergeometric distribution test was used to test for statistically significant overrepresentation of genes from particular biological gene sets within the co-expression in each module and HPV status. The p-values were corrected for multiple testing (FDR-adjusted ≤0.001) using the R package ClusterProfiler^68^.

### Validation with independent microarray and methylation datasets

We analyzed three independent biological datasets, one derived from microarray and two from methylation analyses, downloaded from Gene Expression Omnibus (GEO). For the microarray experiments, we evaluated the GEO: GSE6791^26^, selecting a total of 56 HNSCC samples of which 16 samples are HPV+ (we excluded the cervical samples from the original experiment). We applied the MAS5 normalization method followed by moderated t-tests (HPV+ *versus* HPV−; FDR-adjusted p-value ≤ 0.05 and absolute-logFC ≥ 1) implemented in the affy and limma R packages, respectively^23,69^. For the methylation datasets, we retrieved the experiments deposited on GEO: GSE38266^27^ and GEO: GSE95036^17^, both of which using the 450 K platform. The first dataset consisted of 11 samples (six HPV+) from fresh frozen biopsies. The second one contained 42 samples (21 HPV+) from formalin-fixed paraffin-embedded (FFPE) tissues. We applied the same methodology described for methylation (see in section *Omics datasets and processing*) with absolute-logFC ≥ 1.5 and FDR-adjusted p-value ≤ 0.05.

## Electronic supplementary material

Supplemental Materials

## Data Availability

The networks generated in the analysis above are available in an interactive module at: https://quelopes.github.io/files/projects/HNSCC/Co-expressionHNSCC.html. The HPV+ networks were modeled and populated in the graph database Neo4J. The database can be retrieved at GitHub https://github.com/quelopes/HNSCC-network.
