# Supplementary material for: Distinct co-expression networks using multi-omic data reveal novel interventional targets in HPV-positive and negative head-and-neck squamous cell cancer
Source: Sci Rep. 2018 Oct 15;8:15254. doi: 10.1038/s41598-018-33498-5 (PMC6189122; doi:10.1038/s41598-018-33498-5)
Supplement: Supplementary file 1 — Supplemental Materials [file 41598_2018_33498_MOESM1_ESM.pdf]

SUPPLEMENTAL MATERIALS

Distinct co-expression networks using multi-omic data reveal novel interventional targets in HPV-positive and negative head-and-neck squamous cell cancer

Raquel L. Costa[1,2], Mariana Boroni[2], Marcelo A. Soares[1,3]

Author Affiliations:

[1]Programa de Oncovirologia,Instituto Nacional de Câncer, Rio de Janeiro, Brazil
[2]Bioinformatics and Computational Biology Lab, Instituto Nacional de Câncer, Rio de Janeiro, Brazil
[3]Department of Genetics, Universidade Federal do Rio de Janeiro, Rio de Janeiro, Brazil

#Corresponding Authors: quelopes@Incc.br

Conflict of Interest: The authors have declared that no conflict of interest exists.

This file includes Supplementary Figures 1-7 and Supplementary Tables 1 and 2

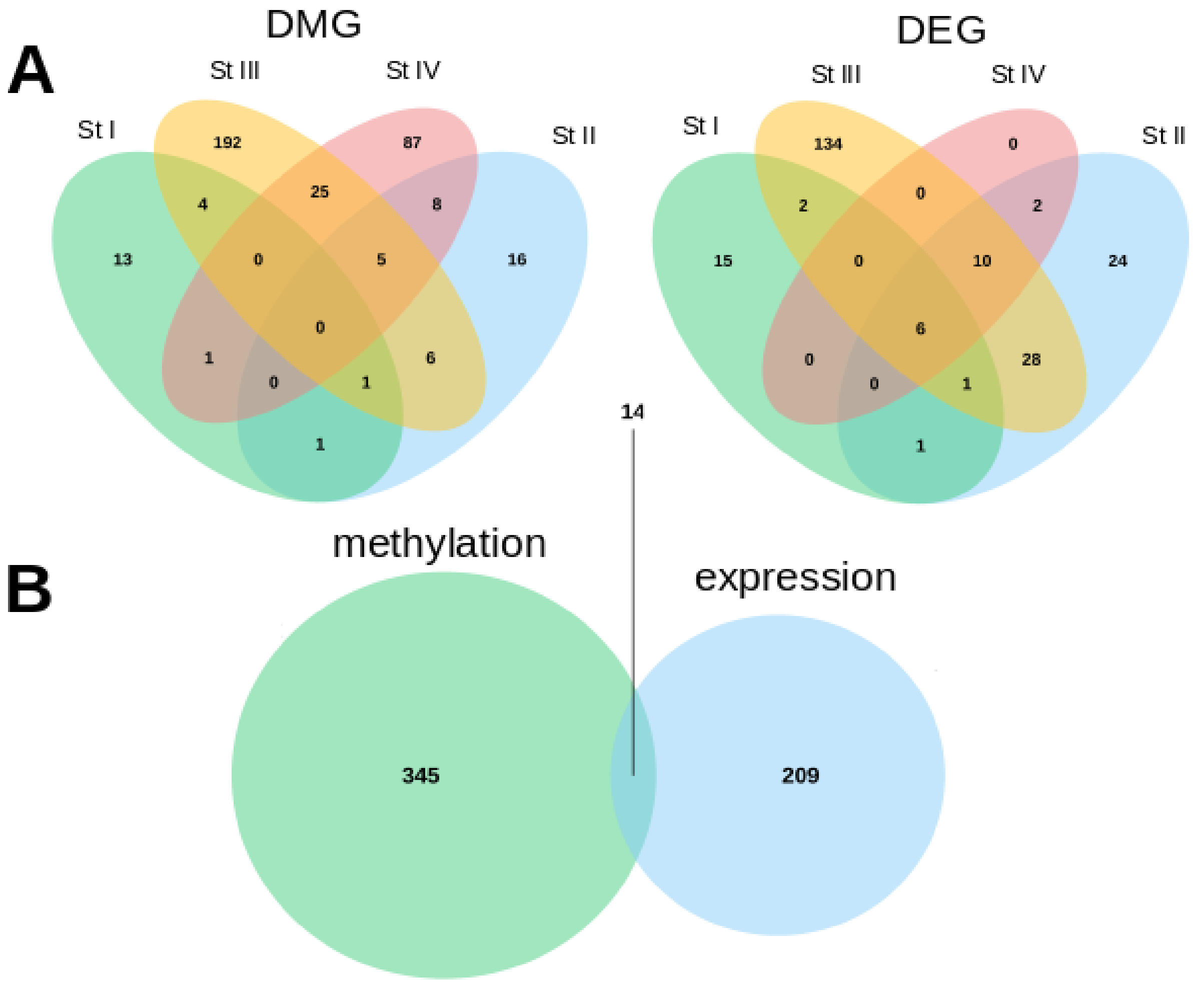

Supplementary Figure 1. In (A) Venn diagrams showing the number of selected genes with differential methylation (DMG; left panels) and expression (DEG; right panels) profiles between HPV+ and HPV- tumor samples. DEG and DMG are stratified by tumor stages (st I to IV, represented by distinct colors), respectively. In (B), the overlap of genes selected in A by differential methylation and expression among HPV+ versus HPV- is shown.

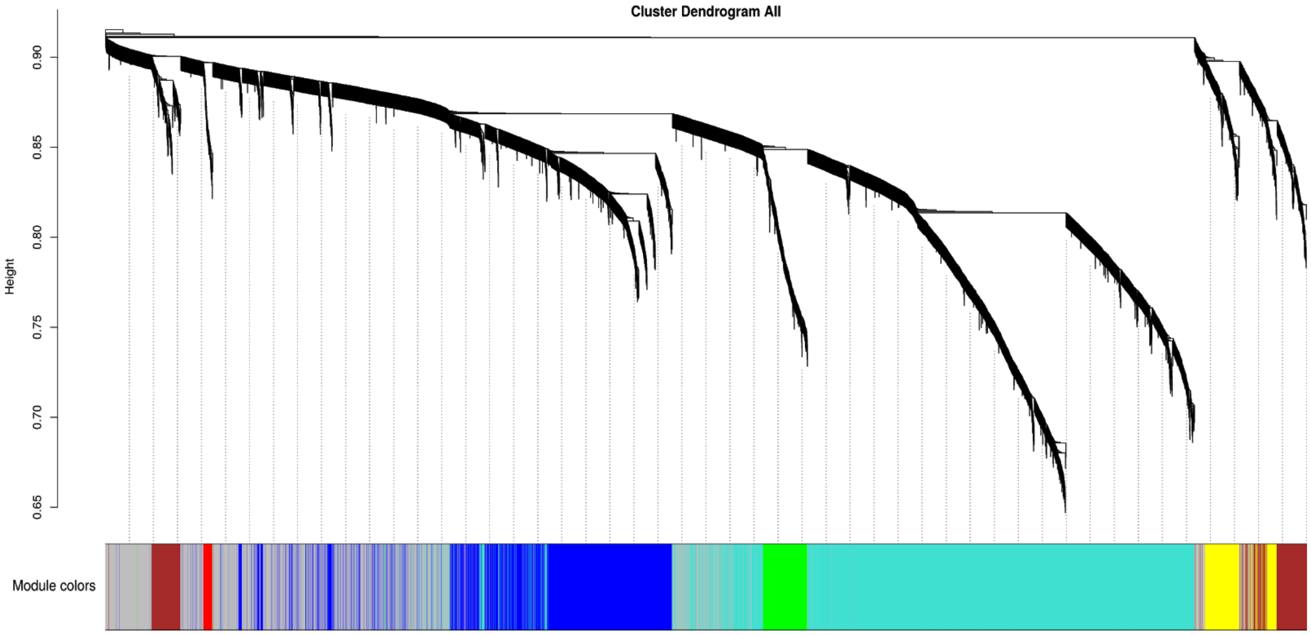

Supplementary Figure 2. Gene clustering dendrogram showing co-expressed genes hierarchically clustered into modules, which were defined by different colors (rectangle at bottom).

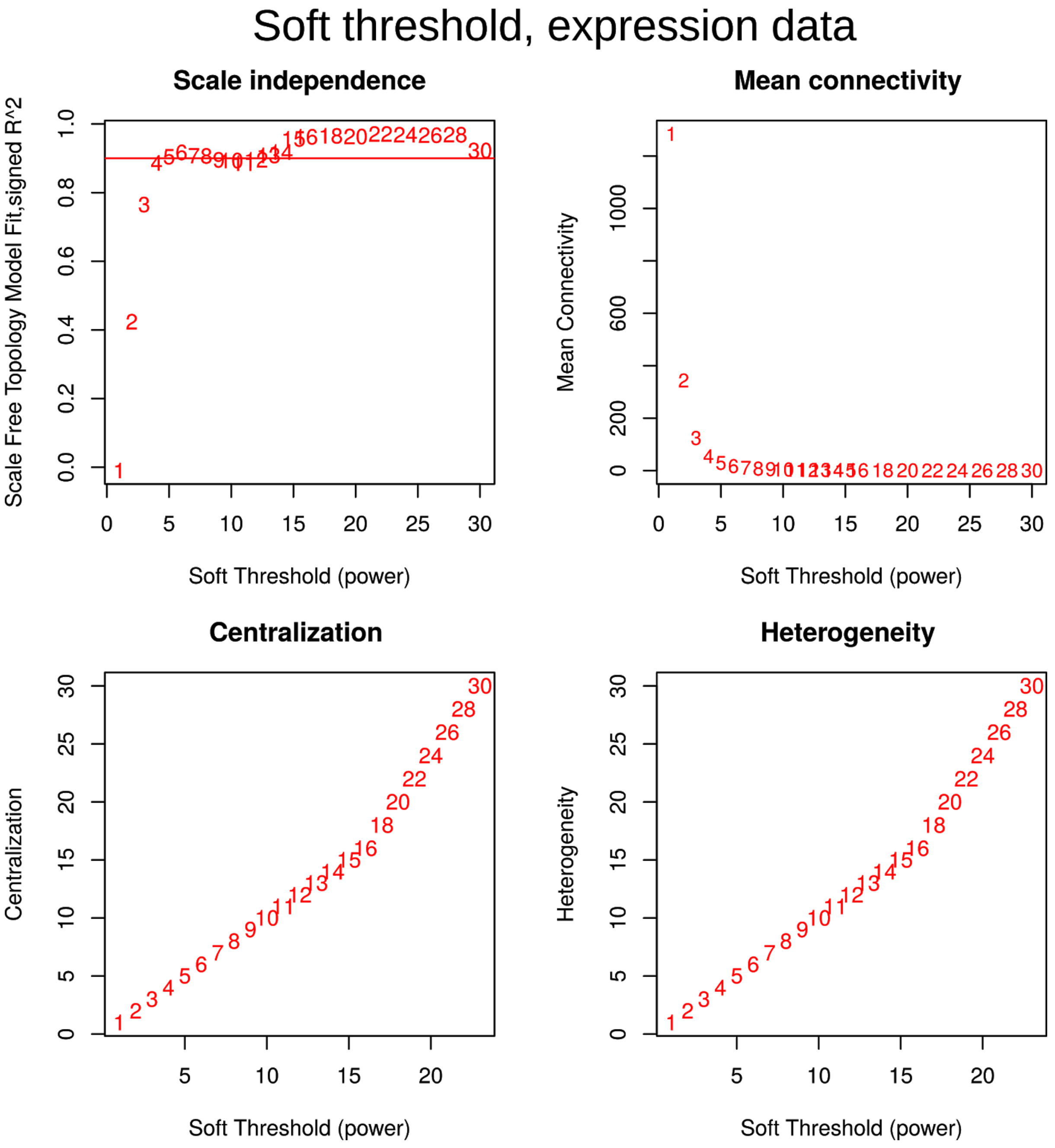

Supplementary Figure 3. Scatter plot of network topology analysis using a range of soft-thresholding powers. Top left, scale-free fit index as a function of the soft-thresholding power indicating the soft-thresholding power of 4. The other charts represent the mean connectivity (top right), centralization (bottom left) and heterogeneity (bottom right) (*y*-axes) *versus* soft-thresholding power (*x*-axes).

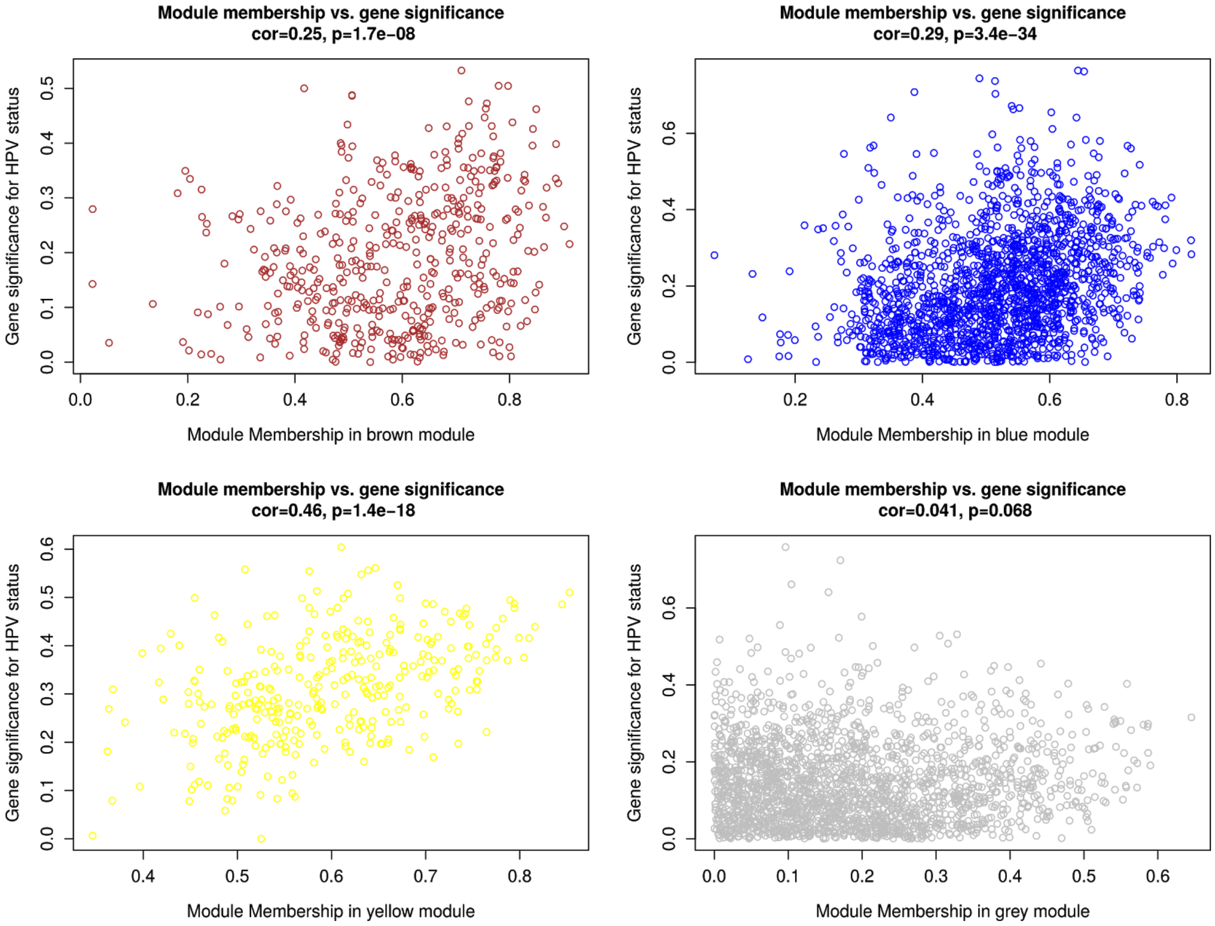

Supplementary Figure 4. Scatter plots of gene significance (*y*-axis) versus module membership (*x*-axis) for HPV-status associated with selected modules with the largest significance when comparing HPV+ and HPV- samples (*Brown, Blue, Yellow* and *Grey*).

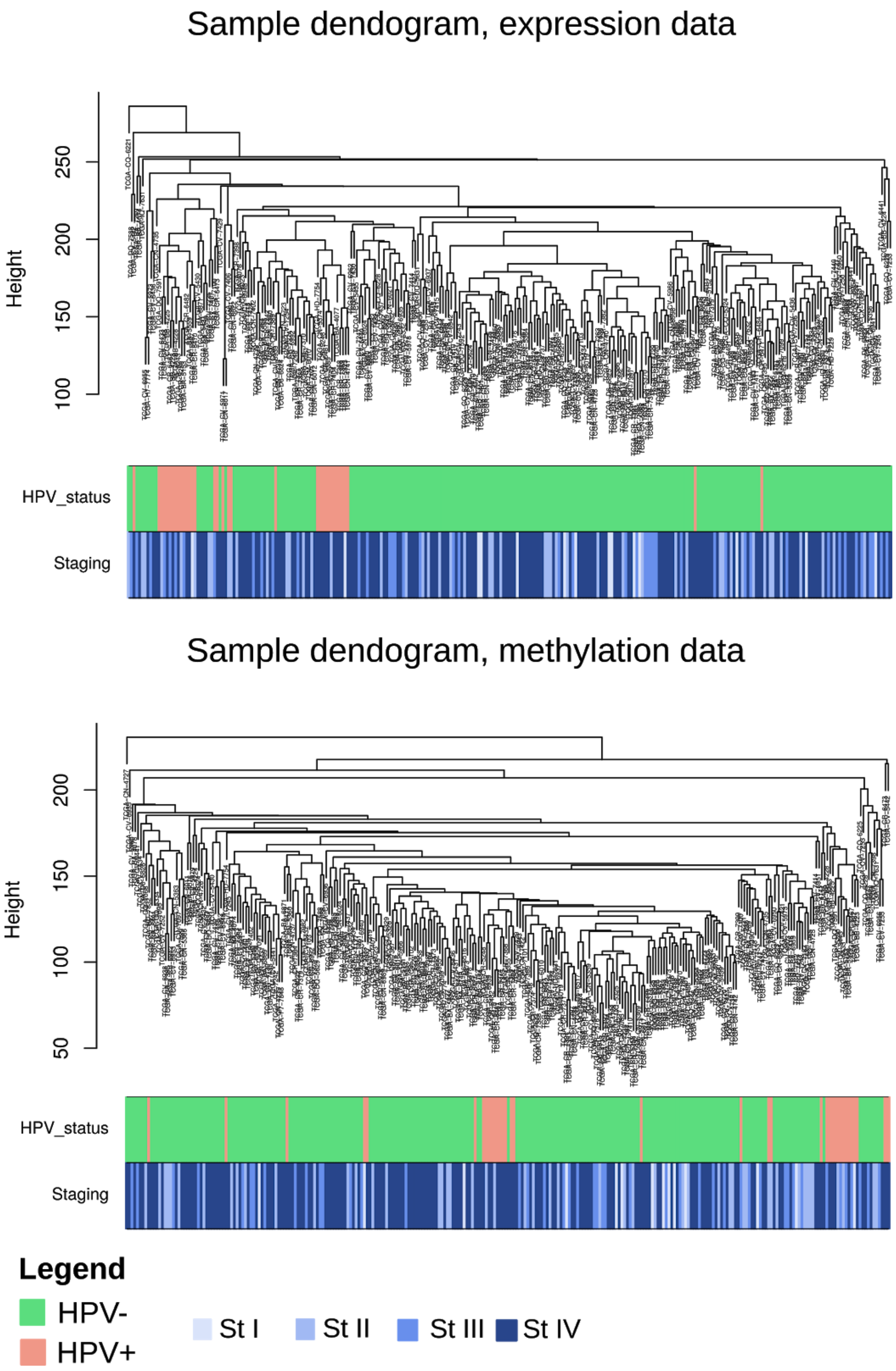

Supplementary Figure 5. Hierarchical clustering of the expression (top panel) and methylation (bottom panel) data of the samples with respect to HPV status or disease staging (horizontal bars at the bottom of each dendrogram, color-labeled according to the legend at the bottom of the Figure.

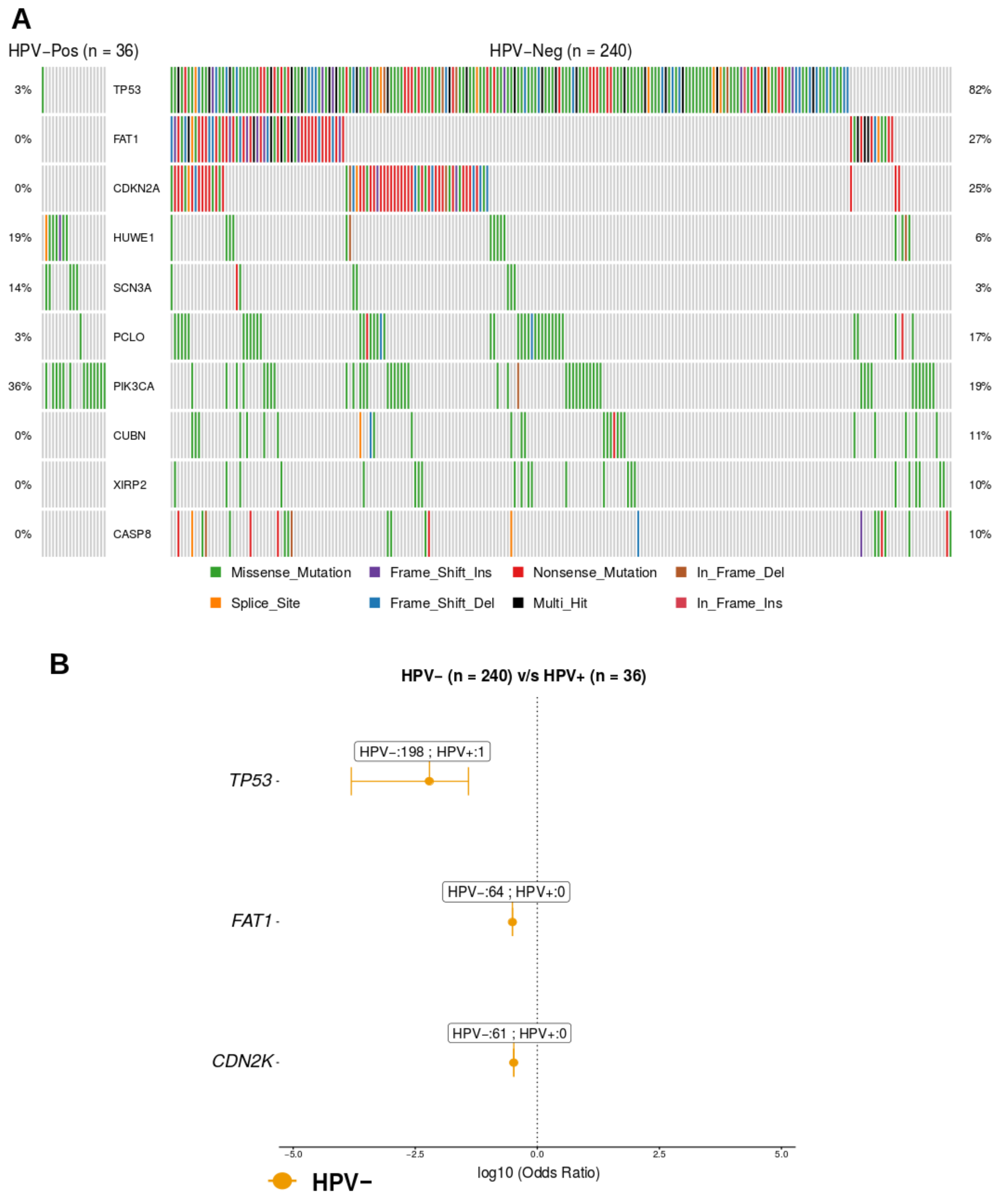

Supplementary Figure 6. (A) Top 10 frequently mutated genes in HPV+ (n = 36) and HPV- (n = 240) HNSCC cases, sorted according to their frequency in the latter. Each case is represented by a single column in both the HPV+ and HPV- graphs. Relative mutation frequencies are shown at the left of the HPV+ graph and at the right of the HPV- graph. Types of mutations found in each gene were color-coded according to the legend at the bottom of the Figure. (B) Forest plot showing the odds ratio (OR) of the significantly mutated genes using the Fisher's exact test (FDR-adjusted $\leq$ 0.05). Only three genes (*TP53, FAT1* and *CDKN2A*) were retrieved with significance, all found in HPV- cases. Values are plotted in a log10 scale.

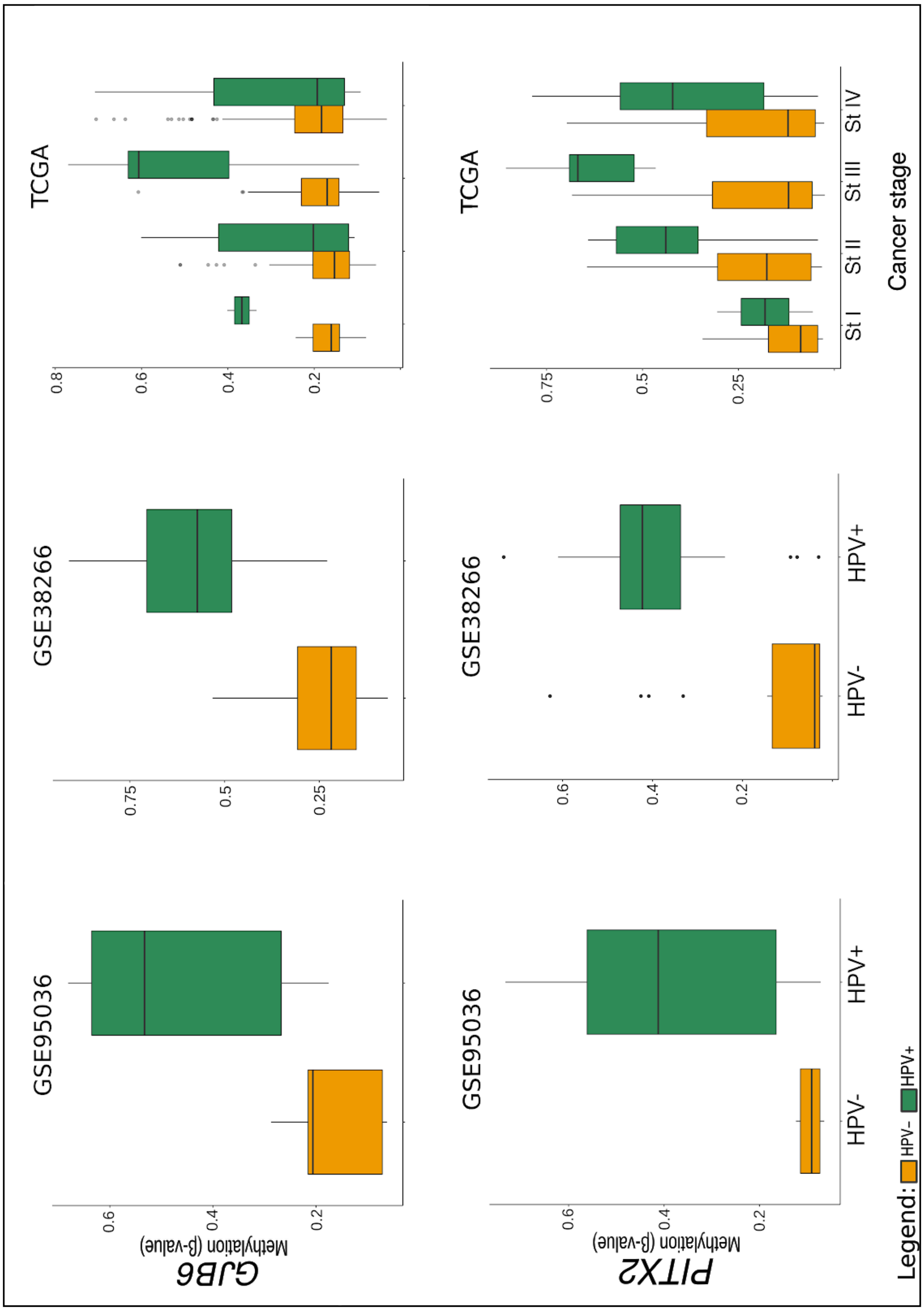

Supplementary Figure 7. Differences in methylation levels of the *GJB6* and *PITX2* gene promoter regions (TS200) between HPV- and HPV+ samples. The left and central panels show data from the external high-throughput experiments deposited under GEO: GSE95036 and GEO: GSE38266. The right panels show the TCGA dataset evaluated in each stage (I--IV). Differences were calculated comparing HPV+ and HPV- samples.

Supplementary Table 1. Differentially methylated and expressed genes (DMG and DEG, respectively) comparing HPV+ and HPV- HNSCC per tumor stage (I to IV). Genes were selected by moderated t-tests, and only those with significant p-values are shown. Adjusted p-values using FDR (≤ 0.01) are also presented. Absolute log fold-change (logFC) values between HPV+ and HPV- are also shown.

DMG_St_I

Genes	logFC	p-values	Adjusted p-values
ZNF442	5.158756126	3.94E-11	2.93E-07
CLSTN1	3.136343742	5.23E-06	0.004789876366
ATP5G2	2.291999017	1.15E-06	0.001547456852
SFMBT2	3.519761261	8.92E-07	0.001324891273
RNLS	3.640967357	6.08E-08	0.0001291392984
BEST3	2.300476142	1.59E-06	0.001814231139
DLC1	4.594633477	5.48E-06	0.004789876366
MC4R	3.785723484	3.75E-07	0.0006188692666
MEI1	-2.670095559	3.92E-06	0.003886294066
ZNF417	4.89254455	7.14E-08	0.0001326125334
CDH26	-2.761310628	6.37E-06	0.005261475335
SIPA1L2	-3.850375945	2.62E-06	0.002783699841
C2orf74	4.343065216	2.26E-10	1.12E-06
ALDH7A1	4.574776481	1.44E-06	0.00178452425
ZNF708	4.127774578	9.91E-06	0.00775022812
ZNF563	5.732553649	5.75E-12	8.54E-08
INPP5F	-2.022160418	1.27E-05	0.009433636238
ZNF530	2.81449348	2.76E-08	6.83E-05
ZNF443	3.102004449	2.10E-08	6.25E-05
PNLDC1	-4.767805931	1.46E-09	5.41E-06

DMG_St_II

Genes	logFC	p-values	Adjusted p-values
PRR5L	-2.149719898	3.83E-09	1.14E-05
STC1	3.083085926	4.67E-05	0.009468975543
C14orf162	-3.12341548	2.54E-07	0.0003436654306
ACSM1	2.461732986	1.90E-06	0.001554514968
OR6K2	2.200172572	3.62E-05	0.00861396541
ZNF772	3.318661585	2.22E-06	0.001650595534
SERINC4	-2.97411914	7.78E-06	0.003210739511
C22orf45	-2.912744449	2.70E-05	0.006788846072

ZNF440	2.901419949	2.29E-09	8.53E-06
ZNF211	2.64034814	1.39E-07	0.0002589396622
ZNF773	3.203902445	1.52E-06	0.001462471011
TMCO5A	2.932620845	1.26E-06	0.001340730272
GCNT2	2.102696746	1.19E-05	0.004118946997
RERGL	2.64609432	3.39E-08	7.21E-05
GRAMD4	-4.311599826	3.07E-05	0.007614373913
HSF4	-3.828352066	4.77E-09	1.18E-05
PKD1	-2.208481486	1.89E-05	0.005213174203
GZMA	3.000892832	3.84E-05	0.00865728557
LSAMP	3.622056477	4.19E-06	0.002009104736
DHX40	-2.92570013	2.81E-06	0.001894100302
HIST1H3C	3.563760575	1.49E-05	0.00490934281
ZNF419	2.519081385	6.44E-07	0.0007980057928
COL29A1	2.815664502	3.91E-06	0.002009104736
APOL6	2.506531756	2.10E-05	0.005681644903
MEI1	-3.217606854	5.50E-10	8.18E-06
APOL2	2.003645112	1.67E-06	0.001462471011
LOC375196	-3.462005372	1.59E-05	0.005016294385
DERL3	-4.020534508	4.91E-05	0.009721531514
HIST1H2BE	3.217815736	4.19E-06	0.002009104736
ZFR2	-2.881044495	4.48E-06	0.00207989787
EDIL3	2.504075615	3.00E-06	0.001894100302
C2orf74	2.810332578	3.57E-05	0.00861396541
MMP3	3.492328251	4.32E-05	0.00925029035
TNP1	2.546401604	1.19E-05	0.004118946997
SYCP2	-5.232800781	1.70E-09	8.53E-06
HIST1H2BB	3.864746321	2.03E-09	8.53E-06
GPR150	-3.455379159	3.78E-06	0.002009104736

DMG_St_III

Genes	logFC	p-values	Adjusted p-values
GJB6	2.060642427	4.94E-05	0.003599463279
LOC401463	3.543586023	1.52E-05	0.00157287264
PTPN22	3.371589943	4.36E-05	0.00333672898
GABRA1	2.985312139	7.87E-07	0.0001646450567
RFXAP	2.344236627	3.68E-05	0.002969034373
PRR5L	-2.383109851	2.70E-06	0.0004454329214

FGF10	2.952733565	2.37E-05	0.002243215581
STMN2	2.188439523	3.17E-05	0.002687442878
ZNF536	2.162945864	0.0001542311617	0.008681929142
STC1	4.864331569	2.72E-10	3.37E-07
HTR1E	2.755091675	4.72E-06	0.0006680721691
CTXN2	2.222149707	7.84E-08	2.43E-05
KMO	2.596493738	3.41E-06	0.0005282921161
PDE1B	2.666365075	3.40E-08	1.20E-05
ZNF442	4.030364877	2.66E-10	3.37E-07
ATP8B4	4.344192196	4.27E-06	0.0006226531509
SLC13A1	2.698241575	5.35E-05	0.003844398546
CECR2	3.451577981	2.96E-05	0.002601991861
NRSN1	3.006781304	4.32E-06	0.0006226531509
DKFZp566F0947	4.029351481	0.0001698016129	0.009346006554
KIF6	4.612134351	6.65E-08	2.15E-05
F2RL1	4.240102582	8.91E-07	0.0001720310784
RECK	4.355765487	1.35E-06	0.0002471998968
FRMD5	2.164940959	0.0001371830506	0.008058013105
ZNF441	2.41972046	3.84E-05	0.003036403352
LAIR1	3.80430294	1.86E-05	0.001843484519
NTSR1	4.090850114	8.74E-06	0.001031211187
HSPC157	2.391100594	1.10E-06	0.0002035347247
NEFL	4.973910082	2.25E-09	1.52E-06
PITX2	3.527734568	1.95E-05	0.001921256235
A2LD1	-2.476424	0.0001635725751	0.009070343426
DPPA2	4.010208259	2.95E-05	0.002601991861
TRAM1L1	3.898366223	1.63E-05	0.001644603555
MIR548F5	2.966611336	1.70E-06	0.0003001000126
CRYM	2.630109983	1.83E-08	7.56E-06
OSM	-2.200827573	4.00E-05	0.003128181708
AGPAT4	2.35991063	4.51E-05	0.00336720798
PRR23B	2.112325418	0.0001178155083	0.007264963769
F2R	4.215035496	3.75E-09	2.32E-06
OR6K2	2.985464568	0.0001354932442	0.007990337707
ZNF772	3.513197876	2.69E-08	9.75E-06
KRTAP12-3	2.090137942	0.0001539396444	0.008681929142
EMB	3.080349167	1.81E-05	0.001805672489
CPPED1	2.466150822	3.35E-05	0.002766986709

CD80	2.947407997	1.58E-07	4.60E-05
PTPN4	2.66461417	2.98E-05	0.002606089159
LPPR1	2.796137255	2.72E-05	0.002482561804
PCDH15	3.651966734	8.56E-07	0.0001718253607
SCARA5	3.615260084	0.0001476469003	0.00844962235
OR2G2	3.03320272	0.0001331328535	0.00794573227
GLIPR1L1	3.402288284	4.89E-14	7.26E-10
C5AR1	2.114230077	8.18E-06	0.001004048786
KCNF1	2.138535824	7.34E-06	0.0009480713901
KIF2B	2.635519203	1.97E-05	0.001923803582
HOXC11	4.453442362	3.82E-05	0.003035819807
FBXO16	2.989205925	5.97E-05	0.004207530309
DYNC1I1	2.742034394	9.18E-07	0.0001749585973
PAG1	3.052975134	1.42E-09	1.17E-06
SLCO1C1	3.432934305	1.37E-05	0.001435878717
UGT8	4.300412615	9.78E-06	0.00113530489
SCGN	2.563194089	1.53E-05	0.00157287264
PAM	4.14672067	6.06E-11	1.29E-07
KIR3DX1	3.116702707	8.39E-07	0.0001708387864
SEMA3E	6.026272065	2.71E-13	2.02E-09
GREM2	4.233889373	5.97E-08	1.97E-05
GCNT2	2.485030127	2.90E-05	0.00259986523
PDX1	4.439952727	3.27E-05	0.00272778453
C19orf45	4.035975666	2.20E-08	8.40E-06
TOX2	2.449314504	8.69E-07	0.0001720310784
IL18	3.116372518	7.28E-05	0.004985763847
GRAMD4	-5.241043467	7.77E-07	0.0001646450567
ABCC13	3.694943658	0.0001140627875	0.007092414582
SLC38A11	4.111187036	3.51E-05	0.002879336776
C17orf77	3.135242285	4.50E-06	0.0006423550929
PLXNA1	-4.142020011	1.04E-06	0.0001959489033
FAM19A2	3.222387151	8.56E-10	8.48E-07
CHST8	3.51315117	3.01E-05	0.002619161139
ZNF273	2.209202915	1.75E-06	0.0003066568872
HSF4	-3.627813191	1.36E-08	6.14E-06
ARPP-21	3.141534669	1.25E-08	5.83E-06
ZDBF2	2.979754605	2.02E-07	5.28E-05
C16orf45	2.61272456	6.21E-06	0.0008245601514

PRMT8	3.579602183	0.0001496167667	0.008486468587
MIR1323	2.238976791	1.39E-05	0.001449645895
FAM169A	3.494545012	8.64E-06	0.001026781706
FLRT3	3.553995318	2.54E-09	1.64E-06
TACC2	-3.542707774	1.55E-06	0.000277055097
HGF	3.19227321	1.16E-05	0.001298562336
PCDH18	2.328625194	4.33E-07	0.0001006087877
SLC6A5	3.445073518	7.49E-08	2.37E-05
PRDM5	3.183293118	2.62E-07	6.60E-05
SULF1	4.567926798	1.80E-09	1.40E-06
MUL1	3.341319051	4.80E-06	0.0006734258067
MIR1265	2.181846302	4.46E-05	0.003343764555
LPL	2.666197796	5.16E-06	0.0007031246919
ADAM5P	2.574432513	1.56E-05	0.001586101428
ZSWIM2	3.776978898	1.49E-10	2.22E-07
LOC643719	3.885406546	7.42E-06	0.0009480713901
FLJ26850	5.400331707	1.54E-07	4.56E-05
CNTN6	2.793315418	0.0001228646484	0.007483162051
ZNF233	3.67632683	2.08E-08	8.12E-06
ZNF30	4.533500822	7.80E-10	8.28E-07
BTBD11	3.972946228	8.29E-09	4.11E-06
DSCAM	2.56061014	2.14E-05	0.00206216941
HTR3A	2.036685499	3.09E-05	0.002651703465
GJA5	2.556595846	3.57E-06	0.0005476624805
RFTN2	3.035385853	2.80E-06	0.0004520136601
LOC169834	4.341641312	0.0001303525544	0.007842790733
CD44	2.279977434	1.86E-07	5.01E-05
ROBO1	3.101059151	1.68E-05	0.001688557427
TRDMT1	3.664544289	7.46E-06	0.0009480713901
COL29A1	2.815868674	1.19E-05	0.001324619121
GPC5	2.850740641	9.12E-05	0.005943737221
NGF	4.456141676	1.27E-09	1.11E-06
ZNF528	3.504554939	0.0001025162538	0.006510658325
ZNF716	3.497419654	0.0001600922777	0.008944102779
TCTEX1D1	2.08734779	0.000159625766	0.008944102779
SYNPR	3.186008427	7.94E-05	0.005365408446
ZNF788	4.324966412	3.17E-05	0.002687442878
SLC17A6	3.35789639	3.88E-05	0.003053587111

LOC283404	2.305067473	9.83E-05	0.006296525491
DIRAS2	2.871123817	2.76E-05	0.002482561804
UGT3A2	2.92240738	2.70E-05	0.002482561804
OLFM3	2.229192474	6.63E-05	0.004645547875
HOXB7	4.822217236	8.26E-06	0.001006142412
SEMA3A	2.117509743	5.52E-05	0.003922289821
MC4R	3.233152242	7.89E-06	0.0009935304087
ZNF552	2.714170177	2.86E-07	7.08E-05
MEI1	-2.417503705	2.43E-05	0.002273692671
HCG11	2.372323187	9.99E-06	0.001151007006
CCL23	3.367405888	1.08E-05	0.001217629604
KCNA1	3.552686136	7.96E-06	0.0009939054388
HPSE2	2.136429471	4.10E-05	0.003177020831
CLSTN2	2.92615083	4.93E-06	0.0006848068851
SPRR2G	2.471534745	6.68E-05	0.004659941373
NEUROD6	2.494335953	4.42E-05	0.003339903984
DNAH9	5.29032835	1.39E-10	2.22E-07
CST7	2.465675337	0.0001063129283	0.00666631404
IDO2	3.369791712	8.29E-05	0.005572962145
KCNJ3	2.961780258	1.08E-05	0.001217629604
EFCAB1	2.087287784	2.96E-05	0.002601991861
GHR	3.521200878	6.76E-05	0.004674027855
PCDHA1	3.503648731	0.0001168200056	0.007233592095
SPOCK1	3.329025369	1.09E-09	1.01E-06
HERC6	2.593990702	4.69E-08	1.62E-05
CADM2	3.009113315	0.0001054242556	0.006647309223
LUZP2	2.775031385	0.0001493698337	0.008486468587
RSPO2	3.145170098	4.43E-05	0.003339903984
LOC400940	3.147051574	8.87E-05	0.005803657868
ZNF610	3.357465194	2.75E-05	0.002482561804
MIR128-2	2.75269963	2.43E-05	0.002273692671
DMRT1	3.120581644	0.0001754735191	0.009522948106
ERMN	2.624359208	1.82E-06	0.0003144469464
LRRC4C	2.183306168	0.0001456463719	0.008389343923
LRRK2	2.954191139	8.13E-07	0.0001677885752
LIPE	3.801500093	9.30E-05	0.006035226929
XIRP1	2.644145311	8.42E-05	0.005585886024
ZNF114	2.813520937	4.26E-06	0.0006226531509

KLHL14	3.856715872	6.08E-06	0.0008137840933
SCAND3	3.263834191	0.0001437659612	0.00835771836
CNTN1	3.351288445	9.51E-05	0.00614481035
NAT1	3.504231219	7.52E-11	1.40E-07
NR1H4	3.538869143	2.71E-05	0.002482561804
EDIL3	4.184090564	5.24E-10	5.99E-07
OR52R1	3.295751636	8.59E-05	0.005646794358
ZNF727	2.03335818	9.07E-06	0.001061546149
OPCML	2.644743319	4.24E-05	0.003265518801
NKD1	2.58197507	3.95E-07	9.47E-05
CMAS	2.250230471	7.33E-07	0.0001578420131
C21orf130	2.081875414	8.36E-05	0.005572962145
CCDC39	2.56700149	2.09E-09	1.48E-06
PER4	3.446306136	3.65E-05	0.002964337206
MMP3	4.070810153	4.74E-05	0.003504272274
CDH18	3.916661154	2.22E-07	5.68E-05
PCDHB6	3.669483974	5.85E-07	0.0001318262658
RAD21L1	3.173614602	2.65E-08	9.75E-06
CDH10	3.730546281	7.41E-06	0.0009480713901
GAS7	3.773818643	5.36E-09	3.06E-06
MIR149	-2.378544258	4.54E-09	2.70E-06
TMEM200C	2.663179067	3.40E-06	0.0005282921161
PCDHB13	3.386472	5.93E-05	0.004195848152
PHOX2B	3.27120156	3.13E-06	0.0004951613701
CPLX2	2.684534554	6.81E-05	0.004683823805
LGALS2	4.238681957	2.02E-08	8.12E-06
HMP19	4.138464553	9.31E-12	2.77E-08
SNORD116-21	2.793112314	2.89E-06	0.0004615347275
CHAT	2.217509595	5.31E-06	0.0007170296017
MSX2P1	2.91771473	4.24E-07	0.000100101369
CCL3	2.00829752	0.0001746152518	0.009522948106
ZNF709	2.788577373	2.07E-06	0.0003540726852
RHOJ	2.104888257	4.68E-07	0.0001069252575
MIR1178	-2.839845062	1.40E-08	6.14E-06
PAH	3.9596904	4.31E-11	1.07E-07
LMO3	3.867730967	1.64E-07	4.60E-05
STEAP4	3.266139594	2.19E-06	0.0003652641259
GPR139	3.637028357	4.10E-05	0.003177020831

TNR	2.176330731	3.25E-05	0.002726943965
CPNE4	3.828879374	1.01E-07	3.05E-05
RHOU	3.031122589	0.0001290758207	0.007797543785
EPB41L1	-2.796474016	4.29E-06	0.0006226531509
CLEC3A	2.082043856	2.09E-05	0.002030333099
LOC644145	3.074939802	6.78E-06	0.0008916586209
C6orf174	3.654325965	7.17E-09	3.95E-06
NLRP3	2.290094177	1.37E-05	0.001435878717
DPPA3	2.78458962	8.35E-05	0.005572962145
SYCP2	-6.016384499	7.75E-09	3.97E-06
MEF2C	2.227725807	0.0001758351079	0.009522948106
PCSK2	3.621687625	0.0001225795864	0.007483162051
MYOD1	3.435693508	6.04E-07	0.0001340003483
PCDHB8	3.301165626	1.31E-05	0.001402690565
NCRNA00159	2.748130207	0.0001762203573	0.009522948106
ALX4	6.656591349	6.05E-12	2.25E-08
CLVS1	2.881337317	0.0001478300122	0.00844962235
FLRT2	3.614435754	0.0001183470462	0.007267584516
ACSS3	3.735108703	1.06E-05	0.001206848193
ZNF257	2.867125175	8.81E-07	0.0001720310784
LRRC3B	3.972414337	1.96E-09	1.46E-06
ZNF563	3.882426832	1.55E-08	6.57E-06
MRGPRE	2.433871741	5.69E-08	1.92E-05
INPP5F	-2.072016781	4.62E-05	0.003430209832
ZNF675	4.56686822	1.27E-05	0.001379379153
IRAK1BP1	2.349147693	1.22E-05	0.001337679086
LOC150622	2.662451215	2.12E-06	0.0003587056269
PRDM13	3.032173837	2.59E-05	0.002403586738
GABRG2	2.707504333	7.52E-09	3.97E-06
ZNF493	3.812200513	3.76E-05	0.003003022139
SHC3	6.319073355	1.84E-12	9.12E-09
OR4K2	2.149792352	3.33E-05	0.002762530215
RUNX1T1	3.618074845	3.64E-06	0.0005517988521
OR10G4	2.591881025	0.0001383701806	0.008095745096
CDH12	4.008889091	3.66E-07	8.91E-05
REG1P	2.674228768	8.39E-06	0.001013306963
ABCD2	2.714249015	1.62E-07	4.60E-05

DMG_St_IV

Genes	logFC	p-values	Adjusted p-values
SPAG17	2.288909076	4.49E-06	0.0002436883657
LIME1	-2.540170877	1.51E-10	6.58E-08
C14orf162	-2.033055407	1.08E-09	3.15E-07
SLITRK3	2.779281279	1.81E-06	0.0001158645755
PRKCB	2.138214	2.34E-05	0.0008853620325
BCL2L14	-2.097099238	1.96E-06	0.0001241072201
GPR98	2.102389744	3.01E-05	0.001097301485
RECK	2.317901054	6.59E-09	1.26E-06
GRM3	2.030704113	4.29E-07	3.68E-05
NTSR1	2.044524324	6.86E-07	5.42E-05
GALM	2.151491752	3.33E-06	0.0001894755105
C7orf58	2.298416788	2.34E-07	2.26E-05
NEFL	2.4198455	1.34E-09	3.62E-07
CDH8	2.190037417	8.41E-07	6.38E-05
RXRG	2.270438199	1.76E-07	1.83E-05
F2R	2.699862853	8.80E-18	3.13E-14
FBXO39	2.064984006	1.80E-05	0.0007153286937
FAM184B	-2.529434088	4.01E-08	5.31E-06
MME	3.479036245	9.10E-09	1.61E-06
NCAM1	2.167568613	5.69E-15	8.45E-12
PAK7	2.702505799	1.26E-07	1.35E-05
SERINC4	-2.205894226	1.26E-09	3.53E-07
FGF12	2.179513333	6.03E-05	0.001832062814
LOC441177	2.265562524	9.21E-06	0.0004238446712
C22orf45	-2.58966434	2.63E-15	4.89E-12
PCDH10	2.031149412	0.0001913440982	0.004194048147
PCDH15	2.117551726	4.28E-10	1.63E-07
ZNF134	2.927540766	1.06E-09	3.15E-07
HOXD1	2.157451538	3.10E-06	0.000178721806
ZNF586	2.089934879	6.77E-10	2.34E-07
ZNF773	2.753062219	7.57E-17	1.61E-13
MYO15B	-2.486222606	3.94E-07	3.48E-05
UNC80	2.461022074	3.63E-07	3.29E-05
KCNV1	2.670038201	1.18E-06	8.29E-05
SSTR1	2.168464003	2.35E-06	0.0001450955431

GREM2	2.945335987	1.53E-11	1.03E-08
PDX1	2.468436613	5.10E-07	4.24E-05
GRAMD4	-4.350767879	1.07E-17	3.13E-14
IRX2	2.319867831	1.56E-08	2.44E-06
CTNND2	2.496414142	9.38E-05	0.002516291301
PCDH20	2.151335421	1.60E-05	0.0006444681743
SLC38A11	2.333548323	2.07E-07	2.01E-05
CHST8	2.000422116	1.92E-06	0.0001223106271
GSX1	2.205000679	1.10E-05	0.0004801997811
HSF4	-3.613307664	1.40E-30	2.09E-26
PRMT8	2.931267848	8.62E-12	6.40E-09
LSAMP	2.035277361	3.94E-06	0.0002184748609
CALB1	2.07482037	1.55E-05	0.0006275705606
IGF1	2.019733706	5.50E-06	0.0002857962079
FOXA2	2.589288541	5.79E-12	4.78E-09
MPDU1	-2.603398234	6.87E-11	3.52E-08
MOCS1	2.31008301	1.80E-05	0.0007153286937
APCDD1L	2.011442845	3.32E-06	0.0001894755105
LOC643719	2.035601236	1.24E-06	8.62E-05
FLJ26850	2.796239338	1.15E-09	3.28E-07
ZNF233	2.241797994	7.76E-15	1.05E-11
DCC	2.099005525	7.03E-06	0.0003438425202
GFRA1	2.64229934	3.86E-06	0.0002148385991
STK32B	2.182546894	0.0001346812544	0.003243919159
ZNF323	-2.289883427	1.31E-06	9.03E-05
RAB3C	2.091088632	4.97E-05	0.001596539354
LOC100192378	2.035450441	0.0004807274152	0.008150919875
ANKRD34C	2.038704609	0.0002361320294	0.004860329762
PXDN	2.099191938	9.60E-05	0.002553273638
GRM1	2.136089038	3.88E-09	8.73E-07
ST8SIA5	2.185884752	5.16E-06	0.000270790369
GRIP1	2.354499159	5.07E-06	0.0002700035102
ZNF788	2.49486807	9.56E-06	0.0004384114588
BSCL2	-2.004937975	6.47E-05	0.00192650203
HYDIN	2.335813156	1.02E-05	0.0004586875988
ANO5	2.262902415	2.27E-05	0.0008667205056
NRXN1	2.274467005	1.06E-09	3.15E-07
STON2	-2.053362826	3.64E-06	0.0002041711381

KCNA1	2.349814746	2.71E-09	6.39E-07
NT5E	2.387359258	8.11E-10	2.74E-07
SFRP4	2.427233276	5.52E-07	4.54E-05
LOC375196	-3.1724186	1.16E-14	1.44E-11
C12orf42	3.013294896	1.22E-10	5.49E-08
DERL3	-3.110517851	4.47E-08	5.83E-06
AJAP1	2.356315188	6.70E-09	1.26E-06
LHCGR	2.238387011	2.14E-08	3.21E-06
MSX2	3.473861692	1.27E-17	3.13E-14
UACA	2.80485812	7.55E-08	8.91E-06
PCDHB1	2.094861829	2.42E-08	3.53E-06
PCDHAC2	2.208584241	5.04E-05	0.001612317191
LOC157627	2.809921822	6.59E-08	7.83E-06
LOC400940	2.033486994	3.65E-09	8.35E-07
ZNF610	2.072890313	8.41E-08	9.77E-06
AMBN	2.099421222	2.12E-06	0.0001322189367
GRIN3A	2.368610889	4.57E-07	3.88E-05
C12orf56	-2.185646275	0.0001009181994	0.002628393899
SLITRK1	2.423154699	4.86E-08	6.12E-06
GCM2	2.368289593	1.99E-06	0.0001250515751
LIPE	2.250294079	6.70E-09	1.26E-06
SYN3	2.083360735	1.56E-07	1.65E-05
EDIL3	2.539134139	3.60E-15	5.95E-12
GABRG1	2.148934087	2.20E-08	3.27E-06
RPH3A	2.290367871	2.43E-06	0.0001486595896
MMP3	2.295378321	5.67E-07	4.60E-05
CDH19	2.480703664	9.54E-11	4.73E-08
CDH18	2.606399553	3.69E-11	2.19E-08
ALDH7A1	2.310159418	6.44E-06	0.0003199763855
PDCL2	2.527921307	1.44E-05	0.0005885849813
CDH10	2.294991742	1.55E-08	2.44E-06
CATSPERB	2.837137294	1.04E-10	4.97E-08
CCNA1	3.093253539	4.81E-07	4.07E-05
SPO11	-2.230567597	5.10E-06	0.0002705733302
SLC22A16	-2.087215359	4.87E-05	0.001566394664
DLEC1	-3.176266942	1.06E-08	1.80E-06
MESP2	-2.480114822	5.63E-08	6.84E-06
C4orf35	2.074867607	1.07E-05	0.0004719870455

SYCP2	-4.515243082	5.35E-21	3.98E-17
PCSK2	3.466644963	3.47E-14	3.96E-11
MIR147	2.200197056	7.92E-07	6.13E-05
GPR150	-2.501442333	2.11E-09	5.21E-07
ALX4	2.683115316	2.22E-12	1.94E-09
ZNF85	2.270169201	1.50E-05	0.0006090253867
CLVS1	2.043053376	4.94E-07	4.12E-05
FLRT2	2.236143334	1.31E-09	3.60E-07
ACSS3	2.216247452	8.99E-07	6.68E-05
PTGFR	2.126920717	5.39E-09	1.11E-06
STAC	2.443151284	4.01E-06	0.0002196503915
CLDN17	-2.139864789	8.56E-08	9.80E-06
SOX30	-2.415440685	6.49E-09	1.26E-06
SGCZ	2.028874905	5.99E-07	4.78E-05
ST8SIA4	3.722250628	9.69E-12	6.86E-09

DEG_St_I

Genes	logFC	p-values	Adjusted p-values
ABCA17P	4.476868797	6.33E-06	0.003943248815
ARHGEF33	4.467217613	1.14E-05	0.006030650822
C11orf85	4.517068171	3.09E-13	3.17E-09
C16orf73	6.845651847	1.16E-09	4.74E-06
C17orf104	4.128009655	4.36E-06	3.19E-03
C22orf45	4.064205793	6.44E-06	0.003943248815
C2orf65	4.735199266	2.78E-05	0.008767662039
COCH	5.411894228	3.00E-05	0.008915184204
FOXE3	4.563062314	1.41E-07	0.0001698640085
GPAT2	6.07098569	5.32E-06	0.003517757231
KIAA1875	4.36841841	1.23E-06	0.001262441357
MMP1	-7.409096364	2.68E-06	0.002290852673
MYO3A	6.154789605	1.42E-05	6.70E-03
NOS2	8.201032795	3.40E-05	0.009853027643
NTS	9.56793497	2.92E-05	0.00881560414
PNLDC1	7.726154025	5.64E-08	7.70E-05
SALL3	7.307314514	3.98E-09	1.16E-05
SMC1B	7.214660791	1.36E-08	3.09E-05
SOX2OT	4.955230539	3.21E-07	0.000365416106
SYCP2	7.328427641	1.86E-10	9.52E-07

TEX15	6.532558103	9.85E-08	0.0001261469344
TLX3	7.168991117	9.79E-23	2.01E-18
UGT8	4.188814321	2.75E-05	0.008767662039
UPB1	4.107798152	1.29E-05	0.006388456547
ZFR2	6.185871277	3.64E-09	1.16E-05

DEG_St_II

Genes	logFC	p-values	Adjusted p-values
ABCA17P	5.86192631	5.98E-14	1.75E-10
ADARB2	4.129046188	7.08E-07	0.000165980055
ARNT2	4.08682466	1.68E-07	5.07E-05
B4GALNT2	4.079242862	1.58E-04	0.00745078627
BTNL9	4.008064864	2.01E-05	0.001881461395
C1orf14	4.359336639	1.67E-18	3.42E-14
C4orf7	6.738705999	8.08E-06	0.0009256807197
CBLN2	4.162211433	3.57E-06	0.000526155433
CCDC155	4.718717309	1.59E-12	2.72E-09
CCL20	4.785931408	2.10E-06	0.0003707378131
CDKN2A	4.756443669	1.62E-04	0.007482588368
CEL	4.397757568	2.20E-04	8.87E-03
CLDN10	5.250078597	4.19E-05	0.003012752186
CLGN	4.027949716	1.43E-04	7.01E-03
COL19A1	4.764812418	9.53E-09	4.65E-06
COL4A4	4.289482942	2.90E-06	0.0004798575148
COL4A6	-4.292612923	4.51E-06	0.0006083366205
CPA4	-4.643946531	5.09E-05	0.003493321888
CPEB1	4.588557513	1.10E-09	7.27E-07
CR2	4.253834256	6.98E-05	0.004298070147
CRB2	4.10177764	3.12E-07	8.53E-05
CSMD1	4.526371775	7.61E-05	4.60E-03
CTSE	4.207441091	5.36E-08	2.00E-05
DMRTA2	5.121524402	6.04E-05	3.91E-03
DSG1	-7.203480329	5.76E-05	3.82E-03
ENPP6	4.075941989	8.47E-08	2.80E-05
FAM3B	4.809961036	5.96E-05	0.003889185977
FOXJ1	4.459177056	0.0002426334431	0.009510952615
GRIN2A	5.448855425	2.40E-07	6.94E-05
GRIN2C	4.092039965	8.34E-12	1.22E-08

HOXB13	4.750154768	3.49E-05	2.68E-03
HS3ST4	5.035288928	4.40E-06	0.0005981634302
IL17REL	4.174282793	4.22E-09	2.38E-06
IRX6	4.224056329	2.02E-04	0.008488240946
KCNB2	4.235385867	9.22E-10	6.30E-07
KCNS1	4.535947633	1.94E-04	8.26E-03
KEL	5.337023307	8.23E-11	7.34E-08
KLK14	-5.093888811	2.37E-04	0.009331251309
KLK5	-6.365412035	5.86E-05	0.003862819594
KLK8	-5.338332757	1.79E-04	0.007926142983
KLK9	-4.988392286	0.0002162551208	0.008799945034
KRT14	-5.509780206	8.77E-06	0.0009938906409
KRT75	-5.262445399	8.48E-05	0.004916054131
KRTDAP	-7.386611106	2.09E-05	0.001946946572
LOC254559	5.19145807	2.41E-11	2.60E-08
MAP7D2	5.874662065	7.49E-07	0.0001689656787
MEI1	4.253472433	6.39E-08	2.18E-05
MMP10	-4.233876515	5.14E-05	0.003500654952
MYO3A	6.752937355	3.68E-10	2.90E-07
PAX1	5.295180105	1.27E-06	0.0002558048846
RNF212	4.98849037	2.29E-06	3.94E-04
RORB	4.90296433	4.84E-10	3.55E-07
SIM2	4.117512237	7.40E-08	2.49E-05
SLC7A2	4.314617518	3.72E-06	0.0005433850528
SMC1B	7.236566264	2.63E-16	1.80E-12
SOX30	4.830001157	1.90E-10	1.56E-07
SPIB	4.174886887	6.14E-06	0.0007576810338
STAG3	5.520418899	2.11E-11	2.40E-08
SYCP2	5.32674452	8.78E-11	7.50E-08
TAF7L	5.107754369	6.45E-13	1.20E-09
TCAM1P	5.692694347	9.52E-12	1.30E-08
TCP11	5.581882701	2.08E-09	1.25E-06
TDRD10	4.344824517	4.02E-11	4.12E-08
TEX15	5.743616865	4.34E-08	1.65E-05
TLX3	4.21999266	8.20E-05	0.004845801689
TRPV6	4.087928698	2.33E-05	0.002076158915
UPB1	4.337469225	3.98E-10	3.02E-07
WNK3	4.16138842	5.43E-09	2.86E-06

YBX2	4.598365514	4.32E-06	0.0005946514241
ZFR2	6.421496786	1.75E-14	6.56E-11
ZNF541	6.318882816	1.92E-14	6.56E-11
ZYG11A	4.549989673	1.56E-05	0.001556252984

DEG_St_III

Genes	logFC	p-values	Adjusted p-values
ABCA17P	5.442220516	2.44E-10	1.51E-07
ABCA3	4.418433933	6.67E-05	2.01E-03
ADARB2	4.340776483	6.65E-07	6.86E-05
AKR1B10	-5.376303433	1.25E-07	2.10E-05
AKR1B15	-5.053638943	7.64E-08	1.50E-05
AKR1C2	-5.457694884	7.24E-06	0.0004159089052
AKR1C3	-4.018388997	1.99E-04	4.25E-03
AMY1A	4.824171353	5.01E-06	3.16E-04
ASCL4	4.126741314	3.02E-11	3.09E-08
B4GALNT3	-4.113756869	7.70E-07	7.62E-05
BARX2	-4.602569972	6.25E-07	6.50E-05
BPIL2	-4.645498856	2.82E-05	0.00107404609
BTNL9	5.575512489	3.47E-07	4.39E-05
C10orf99	-4.25274413	6.14E-07	6.45E-05
C11orf85	4.023926567	9.15E-22	6.25E-18
C11orf92	6.846736198	5.37E-09	1.90E-06
C1orf14	4.785481687	3.03E-15	1.24E-11
C3orf15	4.219153457	1.43E-05	0.0006711132194
C8G	4.687234745	9.64E-12	1.65E-08
CASP14	-5.203433417	1.54E-04	0.003601235578
CCDC155	5.624225897	2.62E-21	1.34E-17
CCNA1	-5.330722831	1.27E-05	0.0006140560802
CDA	-4.757116021	4.17E-07	4.91E-05
CDH16	-4.589068321	7.90E-05	0.002264465993
CDSN	-5.944056172	9.30E-06	0.0004865320671
CHRM3	-4.499924807	1.65E-05	0.0007365522045
CLCA2	-4.171977031	2.46E-11	2.66E-08
CLDN10	5.802808537	2.70E-05	0.001038940126
CLDN3	5.713411444	3.17E-06	0.0002280067417
CLGN	5.751047441	3.46E-09	1.31E-06
CNFN	-4.050630614	2.54E-05	9.97E-04

CNGB1	-4.642103076	1.93E-05	8.12E-04
CNTN1	-4.278616539	4.14E-05	0.00141444369
COL19A1	5.275238256	4.11E-06	2.73E-04
COL4A4	4.012552106	4.68E-05	0.001542066872
COL4A6	-5.177458323	2.84E-08	7.46E-06
CPA4	-4.293401682	0.0001956061716	0.0042078931
CRB2	5.606771588	1.04E-06	9.60E-05
CRCT1	-7.220119106	1.63E-07	2.46E-05
CTSE	5.485070673	1.06E-07	1.89E-05
CWH43	-5.787489544	6.05E-05	0.001868104735
CXCL14	-5.354821302	6.93E-07	7.07E-05
DEFB103B	-6.146343729	8.72E-06	4.76E-04
DMKN	-5.279046519	4.95E-09	1.78E-06
DMRTA2	5.195822872	2.65E-04	0.005212717701
DPF1	-4.041394552	1.54E-07	2.39E-05
DSC1	-5.748600197	1.77E-06	0.0001439048327
DSCR6	4.320988964	1.40E-05	6.61E-04
DSG1	-7.552103709	1.08E-08	3.40E-06
EEF1A2	-4.125934068	1.83E-04	0.004022848875
ENDOU	-4.052464889	1.62E-04	3.71E-03
EPGN	-4.681819053	8.85E-06	0.0004782971406
EREG	-4.31336421	5.66E-04	0.008984677848
EYA2	4.157104862	1.35E-04	3.32E-03
FABP5	-4.821829052	1.10E-07	1.93E-05
FAM25A	-6.807120606	1.37E-08	4.19E-06
FAM25B	-4.497503696	1.46E-05	6.80E-04
FKBP6	4.090031956	5.77E-07	6.10E-05
FLRT3	-5.578076852	4.80E-06	0.0003044583114
GABRP	5.263805904	4.06E-04	7.05E-03
GDPD2	-4.63439494	9.26E-06	4.86E-04
GJB6	-5.877927587	6.79E-10	3.76E-07
GOLGA7B	-4.750253262	4.44E-10	2.60E-07
GRIN2A	6.28053594	1.95E-08	5.56E-06
GSDMA	-4.359189432	6.43E-05	0.001956603027
HEPHL1	-5.888481868	3.84E-06	0.0002589532429
HOXC13	-4.721287807	2.20E-08	6.10E-06
HSPB3	-5.083787313	4.58E-07	5.25E-05
IGFL1	-5.618707849	4.15E-05	0.001414715257

IL17REL	6.45861787	1.33E-09	6.01E-07
IL1F5	-4.723368044	1.53E-06	1.29E-04
IL1F9	-4.798169872	6.44E-06	3.78E-04
IL24	-4.817603663	8.05E-06	0.0004508711256
INSM1	4.443235357	3.42E-06	2.39E-04
IRX4	-4.261395922	0.0001480583633	0.003517201051
ISL1	4.210834843	0.0001882563283	0.004118935951
IVL	-4.763589203	4.40E-08	1.00E-05
KCNA2	4.056860013	6.74E-07	6.91E-05
KCNB2	4.027551417	1.19E-06	0.0001062643443
KCNK10	-4.678150893	1.83E-05	0.000788345458
KCNS1	4.288581594	4.79E-04	0.007902893574
KEL	6.639010588	8.40E-10	4.31E-07
KLK10	-5.244222196	6.33E-09	2.13E-06
KLK12	-5.983398448	6.13E-06	3.67E-04
KLK13	-6.046864984	3.66E-07	4.44E-05
KLK5	-8.271873617	9.55E-09	3.06E-06
KLK6	-7.050702511	3.89E-07	4.64E-05
KLK7	-8.21501996	7.45E-10	3.99E-07
KLK8	-7.465904814	5.99E-14	1.75E-10
KLK9	-6.924941546	3.78E-10	2.28E-07
KPRP	-4.699047232	3.18E-05	0.00117957012
KRT14	-6.23599695	5.82E-11	4.97E-08
KRT16	-5.464709344	5.29E-08	1.14E-05
KRT1	-6.190437443	1.19E-04	0.003040740922
KRT23	-4.159282915	0.0001000803192	0.002675415548
KRT6B	-4.186170827	7.08E-08	1.43E-05
KRT6C	-4.197101107	3.04E-07	3.90E-05
KRT75	-5.939424565	3.58E-06	0.0002468269425
KRT9	-4.359876983	1.39E-06	0.0001189739019
KRTDAP	-8.295302587	1.09E-10	8.00E-08
LASS3	-4.632438891	1.51E-11	2.21E-08
LCE3D	-6.323477244	7.87E-06	4.43E-04
LCE3E	-5.922831539	9.16E-06	0.0004853831326
LOC541473	4.121795055	2.56E-06	0.0001886091413
LY6G6C	-4.652556116	3.23E-05	1.18E-03
MAP7D2	5.069836684	7.38E-05	0.002153349037
MGAT3	4.297482148	2.79E-05	0.001064514852

MMP1	-4.895594764	7.35E-06	0.0004197261765
MMP3	-5.811604494	4.88E-06	0.0003085613783
MYO3A	6.433562252	1.44E-07	2.33E-05
NKX2-3	5.174609844	7.41E-06	0.0004208205332
PAPL	-4.684818518	8.34E-06	0.0004608890873
PAX1	7.753526694	1.90E-10	1.28E-07
PI3	-4.903336178	9.36E-06	0.00048714577
PITX2	-5.700472218	4.16E-08	9.91E-06
PKDCC	4.76502227	4.42E-06	2.88E-04
PLA2G4E	-5.246759138	2.38E-06	1.81E-04
PLA2G4F	-4.418017588	7.68E-08	1.50E-05
PNLIPRP3	-4.905619971	2.50E-04	0.005037393901
PPP2R2C	-4.655894972	9.15E-10	4.58E-07
PRSS3	-5.102085584	6.18E-05	1.90E-03
PTHLH	-5.289140808	2.11E-07	3.01E-05
RANBP17	5.03666534	2.44E-08	6.68E-06
RDH12	-5.530271033	5.77E-07	6.10E-05
RNASE7	-5.343620626	9.79E-07	9.12E-05
RNF128	-4.913059276	7.89E-07	7.74E-05
RNF150	4.011316653	4.26E-05	0.001446086373
RNF222	-4.421638271	9.25E-07	8.78E-05
RORB	5.017985808	1.74E-06	1.42E-04
RPL3L	-4.02929383	1.53E-05	0.0006979758236
S100A12	-5.195681462	7.35E-06	0.0004197261765
S100A7A	-6.974017824	5.20E-06	3.25E-04
S100A7	-8.053876484	3.95E-08	9.63E-06
SBSN	-7.691349899	1.35E-10	9.53E-08
SDR9C7	-5.226885184	2.00E-07	2.87E-05
SIM1	4.801732684	9.72E-10	4.63E-07
SIM2	4.188261278	9.46E-06	4.91E-04
SLC15A1	-4.410915594	0.0001565219653	0.00364503622
SLC35F3	-4.208699678	1.80E-05	0.0007786383807
SLC44A5	-4.09564115	3.64E-07	4.44E-05
SLC5A1	-4.798150184	6.78E-05	0.002029434867
SLC6A11	-5.056611465	1.75E-05	7.68E-04
SLC6A2	-4.059857114	7.08E-05	0.002089641227
SLITRK6	-4.047814166	6.82E-05	2.04E-03
SMC1B	7.822699671	2.29E-23	2.34E-19

SOX30	4.929069726	5.20E-11	4.84E-08
SPIB	5.187326139	1.56E-05	0.0007089151106
SPINK6	-4.797961617	1.70E-04	0.003839028495
SPINK7	-4.849860643	1.57E-04	3.65E-03
SPRR1A	-5.97463794	1.60E-08	4.61E-06
SPRR1B	-6.243222227	7.19E-11	5.89E-08
SPRR2A	-5.06340904	3.54E-07	4.41E-05
SPRR2B	-6.746377089	7.50E-07	7.54E-05
SPRR2C	-4.792738607	1.60E-05	7.21E-04
SPRR2D	-5.942303347	2.23E-09	9.31E-07
SPRR2E	-6.443306001	9.19E-08	1.76E-05
SPRR2F	-5.38579013	6.86E-05	0.002040129764
SPRR2G	-7.314977809	6.23E-08	1.30E-05
SPRR4	-5.972844277	4.63E-06	0.0002963329911
STAG3	6.906119276	5.12E-13	1.31E-09
SV2B	4.047859137	1.57E-04	0.00364503622
SYCE2	4.091409875	1.20E-09	5.61E-07
SYCP2	6.028421208	2.84E-12	5.81E-09
TAF7L	4.846093592	7.59E-10	3.99E-07
TCAM1P	6.445567127	5.49E-11	4.89E-08
TCP11	4.223421286	3.02E-04	5.71E-03
TGM3	-6.688001675	2.25E-04	0.004654820353
TLX3	5.690424432	2.31E-07	3.16E-05
TM4SF19	-4.116597752	1.28E-06	0.0001118047898
UBD	4.674440221	8.89E-05	0.002473616251
UPB1	4.395215922	1.00E-07	1.82E-05
USP2	-4.149671448	3.08E-08	7.89E-06
VCAM1	4.796297205	1.98E-05	0.0008214523026
VSIG8	-4.273370852	0.0005894738022	0.009246941034
WFDC12	-5.62349413	1.05E-05	0.0005284757643
WSCD1	4.054600232	1.11E-07	1.93E-05
XKR4	4.792374526	5.83E-08	1.23E-05
YBX2	4.777904125	1.33E-05	0.0006360010956
ZFR2	6.936356191	1.04E-23	2.13E-19
ZNF541	6.615412146	2.37E-11	2.66E-08
ZYG11A	4.604142616	2.53E-06	0.0001872084898

DEG_St_IV

Genes	logFC	p-values	Adjusted p-values
ABCA17P	4.447484611	6.55E-30	1.92E-26
C4orf7	4.006273623	4.81E-06	8.46E-05
COL4A6	-4.147659185	6.47E-16	2.76E-13
KCNS1	4.154241909	1.70E-11	2.25E-09
KRTDAP	-4.456610894	5.79E-09	3.27E-07
LOC254559	4.056922947	2.80E-20	1.85E-17
MYO3A	4.20439359	1.10E-10	1.05E-08
SMC1B	6.773211239	3.89E-44	3.98E-40
SOX30	4.277430767	9.37E-28	1.75E-24
STAG3	5.477642572	5.85E-38	4.00E-34
SYCP2	5.288044496	2.41E-29	6.17E-26
TAF7L	4.118926744	1.24E-25	1.70E-22
TCAM1P	4.95186132	1.63E-26	2.39E-23
TCP11	4.754093097	3.73E-24	3.83E-21
UPB1	4.079215098	2.54E-36	1.30E-32
YBX2	4.159194322	7.69E-16	3.15E-13
ZFR2	6.248095126	4.75E-57	9.74E-53
ZNF541	5.065092752	8.90E-29	1.82E-25

Supplementary Table 2. Differentially methylated genes (DMG) comparing HPV+ and HPV- HNSCC as defined in the studies by Lechner et al. 2013 (GSE38266) and by Esposti et al. 2017 (GSE95036). Genes were selected by moderated t tests, and only those with significant p-values are shown. Adjusted p-values using FDR (<0.05) are also presented. Absolute log fold-change (logFC) values between HPV+ and HPV- are also shown. Probe IDs and gene symbols are also presented.

GSE38266

Probe ID	gene symbol	logFC	p-values	Adjusted p-values
cg23614791	ACSS3	2.072612107	5.99E-05	0.0132195963
cg08702413	ALDH7A1	1.939621003	0.002267548036	0.1048079903
cg07402669	APCDD1L	1.871962759	0.0002831138932	0.03266377874
cg13702005	C12orf42	2.438358461	0.0003244330983	0.03534755788
cg15731056	C12orf56	-1.933600908	0.0007475424914	0.05822205942
cg06806969	C14orf162	-1.527128434	3.86E-06	0.002493289313
cg03364108	C22orf45	-2.103850657	0.0008046306636	0.06097023964
cg16328106	C2orf74	2.303234243	0.0005336352863	0.04811909682
cg17495912	CCNA1	2.696814892	0.0001782266359	0.02516712372
cg16503924	CDH18	2.148237377	0.0002447551648	0.03013259341
cg16489926	CDH19	1.646890411	1.30E-05	0.005295235555
cg06831576	CDH8	3.309685076	2.97E-06	0.002057877808
cg24551579	CLSTN1	2.028808636	1.37E-05	0.005325078043
cg04996219	CTNND2	2.995666243	0.0001728749776	0.02470093202
cg11417653	DERL3	-2.729851527	1.22E-05	0.005130336051
cg11499681	DNAH9	2.538623271	1.47E-07	0.0002981046814
cg15716405	EDIL3	2.161200098	0.001119903847	0.07283095618
cg02093112	FBXO39	2.807966654	4.70E-07	0.000635469652
cg02403395	FGF12	1.980719607	0.001329584002	0.08008186721
cg01537995	FLRT2	1.69422199	0.0006636243434	0.05440026621
cg05982485	GABRA1	1.700348236	0.001239753391	0.07684803704
cg14250833	GCM2	1.696323827	0.01272947901	0.2609209965
cg20972453	GJB6	2.335315796	4.17E-08	0.0001223274652
cg12864597	GPR150	-1.932187465	0.0003440888413	0.03680622442
cg18794577	GRIN3A	3.230590592	6.15E-07	0.0007527560476
cg10071824	GSX1	1.653542318	0.000755771068	0.05855231658
cg07816687	HSF4	-2.530610498	4.97E-05	0.01182090957
cg09918510	HYDIN	2.085135844	0.00708901239	0.1938092424
cg08235864	IRX2	2.417927983	0.0001096346312	0.01921878722
cg15638055	KCNV1	1.836381003	2.75E-05	0.008195498157

cg12351433	LHCGR	1.845553809	0.0001042780292	0.0187269947
cg12877251	LOC157627	1.606328042	0.01833743015	0.3112900462
cg11176990	LOC375196	-2.076534035	0.0001154456462	0.01960130423
cg22314787	LOC400940	2.037441763	3.43E-06	0.002266126359
cg12874181	LOC401463	1.503604592	0.002511401688	0.1113072182
cg07599133	LOC643719	2.934343324	8.72E-07	0.000965885238
cg22804236	MEI1	-2.615325388	0.0002647756893	0.03128561842
cg22955973	MME	4.167219005	8.95E-10	1.18E-05
cg07182756	MOCS1	1.857564803	0.0005143421445	0.04693942689
cg12387713	MSX2	2.61583615	3.26E-07	0.0005041203174
cg07116393	MUL1	2.109168267	0.0001437985554	0.0222653542
cg22287064	MYO15B	-1.782487751	0.002633789799	0.1141748935
cg01614020	NEFL	2.186659881	0.0025578789	0.1120980884
cg18201351	NRXN1	1.523574219	0.0003789617183	0.03918050512
cg16639665	OPCML	1.66529862	0.0006773721739	0.05501814076
cg12388309	PAK7	2.403846799	0.0002563505649	0.03056808189
cg27600205	PCDH10	3.048816283	3.66E-06	0.002389515888
cg02613108	PCDH15	1.807637232	1.45E-06	0.001249482246
cg25500080	PCDHAC2	2.725600512	2.28E-08	9.01E-05
cg06899976	PCDHB1	1.727430092	8.87E-06	0.004236633901
cg22504204	PCSK2	1.70453339	0.02475996566	0.3527175132
cg04633225	PDX1	2.28741659	2.73E-05	0.008177071015
cg13458645	PITX2	2.865091071	9.83E-07	0.001025810185
cg00735962	PRKCB	1.530336881	0.002030597453	0.09879605255
cg06029846	PRMT8	2.218898344	0.0001704305332	0.02449242389
cg23330451	PRR23B	1.804582777	6.96E-07	0.0008179804996
cg07136133	PRR5L	-1.614459585	0.001620400654	0.08874740235
cg08261837	RAB3C	1.949078343	1.44E-05	0.005442981338
cg25439973	RAD21L1	2.143371886	0.0002093129331	0.02753539137
cg08661007	ROBO1	1.775667093	1.07E-06	0.001051801042
cg16403344	RPH3A	1.873833416	0.001147413053	0.07372191635
cg13901501	RXRG	2.005881325	1.70E-05	0.006074972462
cg15051226	SCAND3	2.219243984	6.04E-06	0.003284503891
cg26552774	SERINC4	-1.566115048	7.89E-06	0.003850725323
cg16818993	SFMBT2	1.531442636	8.61E-05	0.01679192747
cg26337020	ST8SIA4	2.731806926	8.19E-05	0.01618428103
cg09275869	ST8SIA5	1.767194191	0.0001965973331	0.02666322953
cg24202123	STAC	2.743483341	1.39E-05	0.005325078043

cg10351287	STK32B	2.05409794	0.001267020028	0.07774895628
cg09462808	SYNPR	1.523603797	0.0008546305434	0.06296463698
cg05807991	TRAM1L1	4.357750216	1.00E-09	1.18E-05
cg12642431	UACA	1.620893514	4.94E-06	0.002929417605
cg24938830	UNC80	1.885545464	0.0006393883616	0.05332892821
cg13786089	ZFR2	-1.811137761	9.74E-06	0.004366157965
cg07894352	ZNF233	2.367323412	4.76E-08	0.0001308238173
cg21138752	ZNF323	-2.945188228	3.03E-08	0.0001046773185
cg07873812	ZNF419	1.565674445	0.004055144608	0.1436298957
cg27258025	ZNF528	1.865558973	0.007847546805	0.2057353509
cg25419914	ZNF772	1.727995116	0.0008704479779	0.06323482926
cg22411784	ZNF773	2.17901169	1.72E-06	0.001368365071
cg23216292	ZNF85	1.773630671	0.0002507382945	0.03047225666
cg16516691	ZSWIM2	1.848898167	7.39E-07	0.0008512237563

GSE95036

Probe ID	gene symbol	logFC	p-values
cg09103619	ANKRD34C	2.243467784	0.007093712779
cg09968630	ANO5	2.181750039	0.02800570427
cg01175610	BCL2L14	-1.768987901	0.009237381339
cg11711057	BTBD11	-1.584827012	0.00356615275
cg06664258	C12orf42	1.714757596	0.0155377225
cg18407309	CCL3	1.510279776	0.0003753546061
cg21088983	CLVS1	1.719243767	0.03967376941
cg19613722	DCC	1.819397514	0.01967908319
cg25940946	DERL3	-2.539761699	0.01155486888
cg20993403	EPB41L1	-2.309056992	0.02053387303
cg02047489	F2R	1.509755589	0.04844104318
cg27207796	FAM184B	-2.604689997	0.0007428680546
cg27397787	FLJ26850	2.159494157	0.005145205793
cg26597242	GABRA1	1.961896957	0.003118654157
cg18672939	GFRA1	2.184793311	0.01420208545
cg01446515	GJB6	2.238303431	0.01024549576
cg20086208	GPR139	1.810825873	0.03857401694
cg16617543	GRAMD4	-1.654585616	0.04299664737
cg04539573	GREM2	1.7289541	0.03858189245
cg18794577	GRIN3A	2.165037741	0.03858073778

cg14168923	GSX1	1.755302031	0.04344901041
cg01665823	HIST1H2BE	-1.536629414	0.03342192421
cg03472798	HTR1E	2.028230266	0.009863346266
cg08208133	HTR3A	1.873064413	0.0008607513607
cg16342115	KLHL14	2.224785601	0.007528256744
cg19403014	LHCGR	2.351857819	0.009663120844
cg15248835	LOC157627	2.094657825	0.008390325723
cg24603926	MIR1178	-1.723409588	0.03136116474
cg12387713	MSX2	2.041971535	0.01915717275
cg15138846	NAT1	1.505683895	0.04021881425
cg07733800	NCAM1	1.530103918	0.006044332722
cg12832313	PCDHAC2	2.145016613	0.01094884554
cg26731781	PDX1	2.103535388	0.01798173473
cg16002355	PITX2	2.343803349	0.01716874399
cg00735962	PRKCB	1.739552777	0.02827761898
cg13177747	RSPO2	1.559876001	0.02346895508
cg22675486	RXRG	3.348253641	0.0001581276535
cg27136241	SCAND3	2.409292401	0.01602879697
cg16818993	SFMBT2	2.017231643	0.02357684253
cg06804921	SLC6A5	2.178957195	0.001978004017
cg22214414	SYCP2	-2.064359518	0.002388437366
cg06314761	SYNPR	1.707162612	0.01950292076
cg11824639	TCTEX1D1	1.747188785	0.02539177362
cg07908508	ZNF30	1.643590377	0.01724048749
cg23989004	ZNF709	1.538647484	0.002561627319
cg21117673	ZNF773	1.692732298	0.03060082021
